# Slow unloading leads to DNA-bound *β*_2_-sliding clamp accumulation in live *Escherichia coli* cells

**DOI:** 10.1038/ncomms6820

**Published:** 2014-12-18

**Authors:** M. Charl Moolman, Sriram Tiruvadi Krishnan, Jacob W. J. Kerssemakers, Aafke van den Berg, Pawel Tulinski, Martin Depken, Rodrigo Reyes-Lamothe, David J. Sherratt, Nynke H. Dekker

**Affiliations:** 1 Department of Bionanoscience, Kavli Institute of Nanoscience, Faculty of Applied Sciences, Delft University of Technology, Lorentzweg 1, 2628 CJ Delft, The Netherlands; 2 Department of Biology, McGill University, Montreal, Quebec, Canada H3G 0B1; 3 Department of Biochemistry, University of Oxford, Oxford OX1 3QU, UK

## Abstract

The ubiquitous sliding clamp facilitates processivity of the replicative polymerase and acts as a platform to recruit proteins involved in replication, recombination and repair. While the dynamics of the *E. coli β*_2_-sliding clamp have been characterized *in vitro*, its *in vivo* stoichiometry and dynamics remain unclear. To probe both *β*_2_-clamp dynamics and stoichiometry in live *E. coli* cells, we use custom-built microfluidics in combination with single-molecule fluorescence microscopy and photoactivated fluorescence microscopy. We quantify the recruitment, binding and turnover of *β*_2_-sliding clamps on DNA during replication. These quantitative *in vivo* results demonstrate that numerous *β*_2_-clamps in *E. coli* remain on the DNA behind the replication fork for a protracted period of time, allowing them to form a docking platform for other enzymes involved in DNA metabolism.

## Abstract (web summary)

DNA replication is accomplished by the replisome, a multi-protein complex that comprises the sliding clamp. Here, Moolman *et al*. present quantitative and dynamic measurements of the number of *β*_2_-sliding clamps at the single-cell level in live *E. coli* cells to shed light on key aspects of DNA replication.

## Graphical abstract

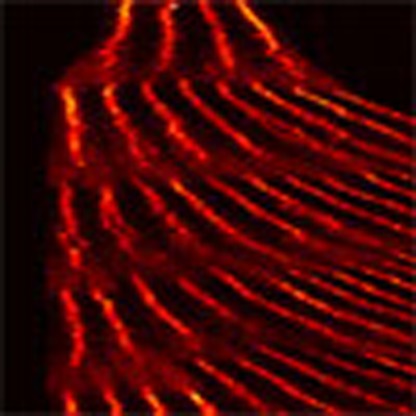

The multi-protein replisome complex (replisome, Fig. 1a) is responsible for the accurate and timely duplication of the genome before cell division. The sliding clamp protein complex is a key subunit of the replisome and is vital for protein–DNA interactions related to DNA metabolism in all three domains of life^1^,^2^,^3^. Through their interaction with polymerases, DNA ligase, replication initiation protein DnaA, the dynamin-like protein CrfC, as well as different mismatch-repair proteins, sliding clamps have important roles in replication and repair^4^,^5^,^6^,^7^,^8^,^9^,^10^,^11^,^12^,^13^,^14^,^15^. In *E. coli*, the *β*_2_-sliding clamp (*β*_2_-clamp) is a homo-dimer^16^ (Fig. 1a (inset)) that encircles double-stranded DNA (dsDNA) and tethers DNA Polymerase III (DNA Pol III) to the template, thereby ensuring sufficiently high processivity during synthesis^17^,^18^.

The *β*_2_-sliding clamp is actively assembled and disassembled onto DNA during the synthesis of the two complementary DNA strands (Fig. 1b). The loading reaction of a *β*_2_-clamp onto each new primer-template junction^19^ is catalysed by an ATP-dependent heteropentameric clamp-loader complex (clamp-loader), also known as the γ-complex^20^. The clamp-loader pries open the *β*_2_-clamp, recognizes the primer-template junction^21^ and closes the *β*_2_-clamp around the dsDNA before release^22^. The clamp-loader is also thought to chaperone DNA Pol III onto a newly loaded *β*_2_-clamp^23^ and to unload inactive DNA-bound *β*_2_-clamps via the δ-subunit^24^. During all of these reactions, the loader complex and the various clamp binding proteins compete for the carboxy (C)-terminal face of the clamp. In accordance with the proposed model in which the replisome includes three core DNA polymerase IIIs^25^,^26^,^27^, three *β*_2_-clamps can be active at the replication fork, one for each of the three polymerases (Fig. 1a). While leading-strand replication is thought to be continuous, utilizing only a single *β*_2_-clamp, the lagging-stand template is copied in discrete 1–2 kb Okazaki fragments^28^, each utilizing a separate *β*_2_-clamp. These fragments are initiated by the continuous formation of 10–12 nt RNA primers by the primase (DnaG), which, together with the helicase (DnaB), sets the replication fork clock^29^. Since the number of Okazaki fragments (2,000–4,000) for the 4.6 Mbp genome is roughly an order of magnitude greater than the average number of *β*_2_-clamps per cell in a nutrient-rich culture^24^,^30^, continuous recycling of *β*_2_-clamps is necessary for total genome replication to occur.

Despite numerous *in vitro* and *in vivo* studies, it still remains unclear whether recycling of the *E. coli β*_2_-clamps takes place immediately following the completion of an Okazaki fragment, or at a later time. A slow recycling could permit a *β*_2_-clamp to fulfil additional functions, while remaining bound to the newly synthesized DNA. Quantitative *in vitro* unloading assays^24^,^31^ indicate that in the absence of the clamp-loader, a loaded *β*_2_-clamp has a long half-life of *t*_unload_>1 h on the DNA. Although this is decreased by more than an order of magnitude to *t*_unload_~127 s per *β*_2_ in the presence of clamp-loader, this unloading time still remains long compared with the typical time required to complete an Okazaki fragment (on the order of seconds). Such a slow unloading time suggests that many *β*_2_-clamps are left behind in the wake of the replication fork^32^. However, a recent *in vitro* single-molecule study indicates that lagging-strand synthesis can persist *in vitro* in the absence of excess *β*_2_-clamps in solution, implying that a *β*_2_-clamp can be directly reused at a successive primer-template junction^33^. Two *in vivo* studies, one in *Bacillus subtilis* (*B. subtilis*)^34^ and the other in *E. coli*^26^, provided contrasting results. Hence, to understand the regulatory mechanism that underlies the recycling of *β*_2_-clamps in *E. coli*, further insights into their *in vivo* dynamics are required.

To gain detailed insight into the *in vivo* recruitment and turnover of the *β*_2_-clamp, we investigate its dynamics in individual live cells with single-molecule sensitivity. We use both conventional fluorescence microscopy and Photoactivated Localization Microscopy (PALM)^35^,^36^, in combination with custom-built microfluidics. Single-molecule techniques have provided us with insights into the dynamics of processes—such as replication, transcription and translation—that are not readily accessible with conventional ensemble-averaging techniques^37^,^38^. *In vivo* single-molecule fluorescence imaging, in particular, has provided detailed insights into the behaviour of individual molecules in live cells^39^,^40^,^41^. Combining single-molecule fluorescence microscopy with microfluidics allows us to image individual molecules in live cells over multiple cell cycles, without chemical fixation that could potentially perturb the dynamic behaviour of the protein under investigation^42^.

By using this experimental approach, we have measured the number of DNA-bound *β*_2_-clamps during chromosomal replication over the entire course of the cell cycle. In addition, we have determined the time required to unload an individual DNA-bound *β*_2_-clamp during replication, as well as the effective time required to load a new *β*_2_-clamp during replication. Our data reveal that the number of DNA-bound *β*_2_-clamps accumulates on the DNA after initiation, and then levels off to a constant steady-state number of DNA-bound *β*_2_-clamps on the order of minutes. This steady state is maintained throughout the rest of the replication process, until termination occurs and a concomitant decrease of DNA-bound *β*_2_-clamps is observed. The number of DNA-bound *β*_2_-clamps in steady state exceeds the estimates of a previously published *in vivo* study^26^ by an order of magnitude. The measured values for the effective loading time and unloading time during replication, in the context of the live cell, are in good agreement with previous biochemical *in vitro* experiments^24^. Taken together, our data indicate that a *β*_2_-clamp remains on the DNA for a protracted period of time following the completion of an Okazaki fragment. DNA-bound *β*_2_-clamps that are left behind during fork progression may facilitate the recruitment of additional proteins active during the cell cycle for different processes such as DNA repair.

## Results

### The *in vivo* dynamics of *β*_2_-clamps measured in single cells

**Figure 2:**
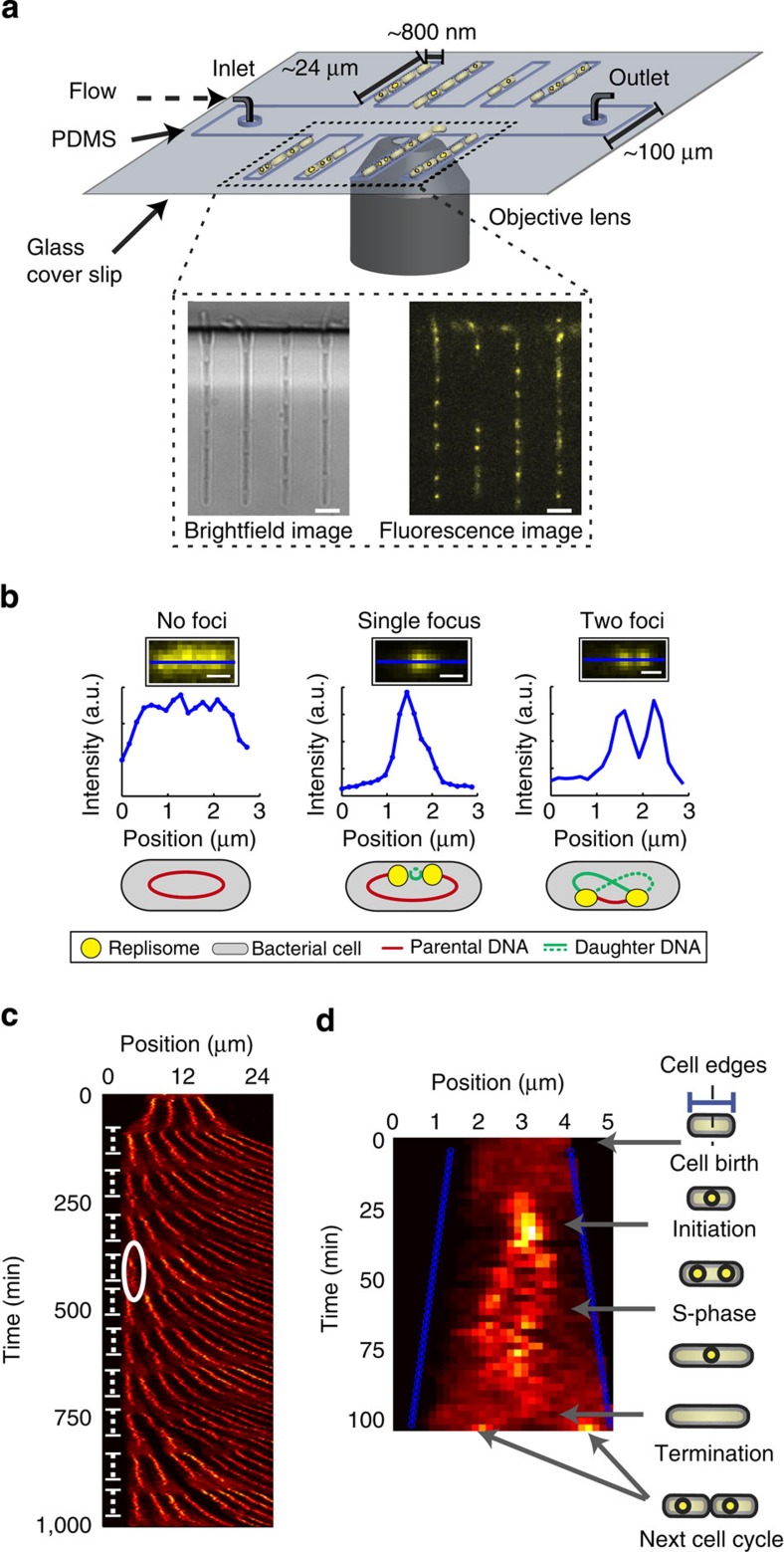
Long time-lapse fluorescence microscopy of the *β*_2_-sliding clamp at the single-cell level utilizing microfluidics. (**a**) The microfluidic device used for performing long time-lapse fluorescence microscopy. *E. coli* cells are immobilized in growth channels perpendicular to a main trench through which growth medium is actively pumped. (inset) A brightfield image and corresponding YPet–*β*_2_ fluorescence image (80 ms laser light exposure) are acquired every 2.5 min for the duration of the time-lapse experiment. Scale bar, 3 μm. (**b**) YPet–*β*_2_ molecules that are either DNA-bound or freely diffusing are studied using wide-field fluorescence microscopy. (left) Freely diffusing YPet–*β*_2_ molecules in the cytoplasm of a cell. This signal is representative to YPet–*β*_2_ dynamics before and after replication. (middle) A clear focus is observed due to DNA-bound YPet–*β*_2_ molecules. The observation of a single focus, instead of two distinct foci, shortly after initiation results from the overlap of diffraction limited spots. (right) Two distinct foci are visible, indicative of two individual replisomes. Scale bar, 800 nm. (**c**) Kymograph of a single growth channel during an overnight time-lapse experiment. The cells first grow the growth channel full, and maintain a steady state growth rate as can be observed from the curved shape of the fluorescence signal. The shape of the fluorescence signal is due to the individual cells growing and pushing each other in the direction of the main trench. Clear observable diffuse patterns occur at regular intervals, indicative of no DNA-bound *β*_2_-clamps before initiation or after termination. This repeating pattern is due to the multiple cycles of replication (indicated with repeating white dashed lines). (**d**) A kymograph of an individual replication cycle indicated in **c**. The blue lines are the cell boundaries detected from the brightfield images. The illustrations on the right-hand side indicate the different stages of replication that can be observed during the cell cycle.

To study the dynamics of *β*_2_-clamps by wide-field fluorescence microscopy, we perform long time-lapse imaging of labelled *β*_2_-clamps over multiple replication cycles. During such experiments, we ensure healthy cell physiology by implementing a custom-built microfluidic device (Fig. 2a; see Methods)^43^,^44^ in which cells growing in steady state are immobilized in micron-sized growth channels. Through a neighbouring central trench, growth medium is continuously supplied throughout an experiment (see Methods). In such a microfluidic device, cells experience minimal perturbation over the course of the time-lapse experiment, as stable growth conditions remain continuously present. This contrasts with long time-lapse experiments performed on agarose pads in which nutrients and water may become depleted, leading to non-steady state cell populations as a result. Additional benefits of such a device are that daughter cells ultimately grow out of the growth channels, preventing the accumulation of cells, and that the cells are always aligned, which facilitates data analysis.

Labelling of the *β*_2_-clamp was accomplished by using a functional amino (N)-terminal YPet^45^ fusion^26^ (Supplementary Fig. 1; Supplementary Table 1; see Methods) expressed from (and replacing) the endogenous *E. coli dnaN* gene locus. Fluorescence images are acquired under shuttered 515 nm laser excitation (see Methods; Fig. 2a (inset)). Fluorescence images of YPet–*β*_2_ within individual cells either yielded no (Fig. 2b, left), a single (Fig. 2b, middle) or two cellular foci (Fig. 2b, right), depending on the stage of replication, in agreement with previous reports of fluorescently labelled replisome components^46^. Before each fluorescence image, a brightfield image is acquired to provide details of the cell periphery (Fig. 2a (inset)). This alternating imaging sequence has a sufficiently long period to avoid giving rise to any notable deleterious growth effects, as assessed by comparing the doubling time of cell growth in a shake flask with cells grown in the microfluidic device (Supplementary Figs 1 and 2).

Using this approach, we are able to observe numerous consecutive replication (and corresponding division) cycles of cells in the different growth channels. We examine the global replication dynamics of multiple cells within a growth channel by converting the time-lapse images into a kymograph (Fig. 2c; see Methods). A distinct reoccurring pattern indicative of multiple replication cycles in the generations of cells is clearly noticeable (indicated by repeating dashed lines in Fig. 2c). Under these experimental conditions (see Methods), the analysis of individual cells (*n*=137) in our microfluidic system yields an average replication time of *t*_rep_=68±10 min, and a doubling time of *t*_double_=84±17 min (Supplementary Fig. 2). In both cases, the error is ±s.d. We further analyse these kymographs to investigate the subcellular dynamics of the YPet–*β*_2_ molecules within the individual cells from cell birth till cell division (Fig. 2d). One can clearly observe the dynamics of the two *β*_2_-clamp foci associated with the two independent replisomes.

### The assembly and accumulation of *β*_2_-clamps on DNA

**Figure 3:**
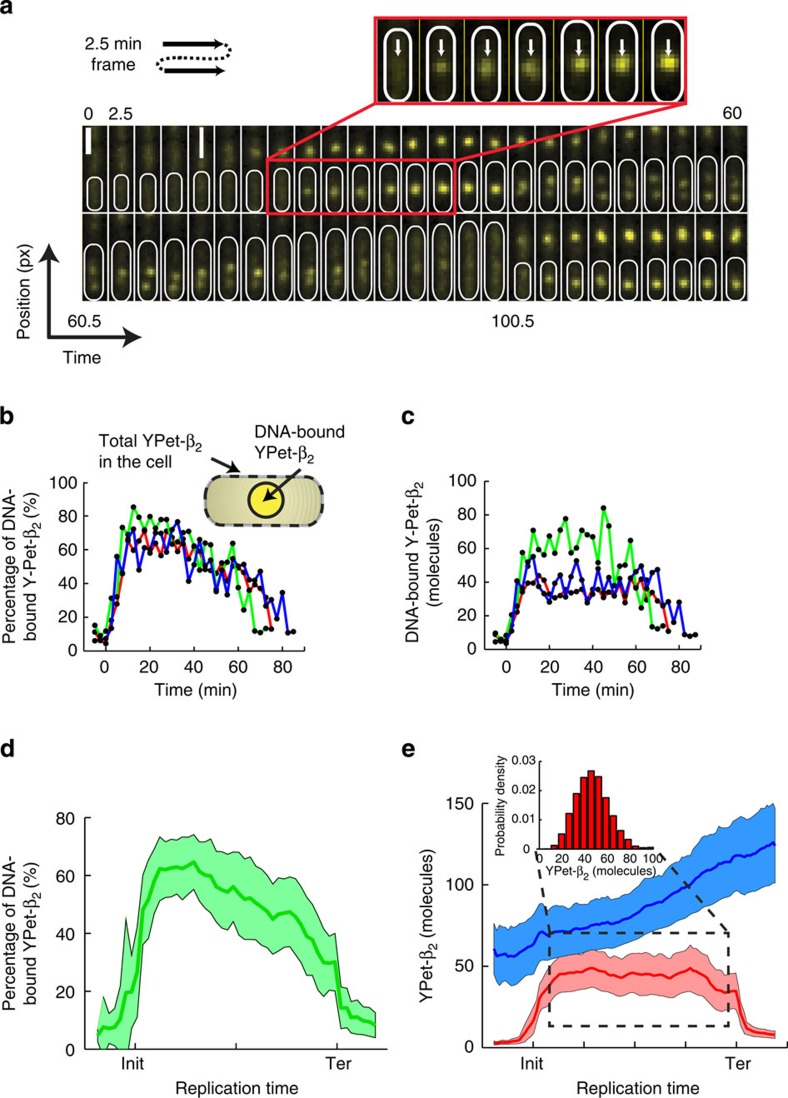
Quantification of the *in vivo β*_2_-sliding clamp stoichiometry during replication. (**a**) A representative temporal montage of the YPet–*β*_2_ fluorescence signal from before initiation until after cell division. A clear intensity increase is observed at the focus formation following initiation (indicated with white arrows in inset) Scale bar, 1.6 μm. (**b**,**c**) Traces of focus formation in individual cells. In **b**, we plot the fraction of YPet–*β*_2_ molecules that are DNA-bound compared with the total number in the cell. More than 50% of the total YPet–*β*_2_ molecules are DNA-bound. The gradual decline in this fraction results from the increase of *β*_2_ during the cell cycle. The inset indicates how the DNA-bound YPet–*β*_2_ molecules and the total YPet–*β*_2_ in the cell are defined. In **c**, we plot the absolute number of DNA-bound YPet–*β*_2_ molecules. Here, the gradual increase, steady state and gradual decrease of the DNA-bound YPet–*β*_2_ molecules can clearly be seen. In both **b** and **c**, the traces have been aligned with respect to initiation. (**d**,**e**) The average behaviour of individual YPet–*β*_2_ molecules measured in individual cells. (**d**) The fraction of DNA-bound YPet–*β*_2_ molecules is on average >50% half way through the replication cycle. (**e**) The YPet–*β*_2_ molecules in the whole cell (blue curve) approximately doubles during the cell cycle, from 60 to 120 YPet–*β*_2_ molecules. The DNA-bound YPet–*β*_2_ molecules (red curve) remarkably increases to a mean steady state value of 46 YPet–*β*_2_ molecules (s.d.=12, s.e.m.=1) following initiation. This value is maintained throughout the replication process until a concomitant decrease is observed after or during termination. Individual traces have been normalized with respect to initiation and termination to make averaging possible. (inset) A histogram of the distribution of number of DNA-bound YPet–*β*_2_ molecules during steady state. (*n*=137).

We use the fluorescence intensity from the YPet–*β*_2_ fusion to determine the number of *β*_2_-clamps that are DNA-bound as well as in the total number in the cell during the life cycle of a cell. A sample montage of the YPet–*β*_2_ fluorescence signal from cell birth till after cell division (Fig. 3a) illustrates that there is a distinct increase in the foci following the B-period^47^ of the cell cycle (represented by a diffuse signal after birth) (Fig. 3a (inset)) and a similar decrease before cell division. The fraction of fluorescence that originates from DNA-bound YPet–*β*_2_ (foci) provides clear evidence that >50% of *β*_2_-clamps are DNA-bound shortly after the initiation of replication (Fig. 3b). The steady decline in the fraction of DNA-bound *β*_2_-clamps that commences roughly 10 min after initiation, results from the increase of total number of *β*_2_-clamps in the cell as the cell grows. In assessing this intensity fraction, we verified that very little out-of-focus fluorescence escapes detection (Supplementary Note 1; Supplementary Fig. 3).

### A constant number of DNA-bound *β*_2_-clamps is maintained

To precisely quantify the number of DNA-bound *β*_2_-clamps as a function of the replication cycle, we exploited a single-molecule *in vitro* calibration method^26^ that allows us to reliably convert the detected YPet–*β*_2_ signal into an absolute number of molecules (Supplementary Note 2). We immobilize single purified YPet molecules on a cover glass and determine the average intensity of a single YPet fluorescent protein under these conditions (Supplementary Fig. 4). Using this calibration, we perform control stoichiometry experiments of previously studied DNA-bound replisome components^26^, specifically the *ε*-subunit of Pol III and the *τ*-subunit of the clamp-loader to verify that our *in vitro* single-molecule calibration remains reliable *in vivo*^48^ (Supplementary Fig. 5). For both the proteins, we reproduced the stoichiometry for the pair of sister replisomes as previously published^26^, namely 5.74±0.04 molecules in total for the *ε*-subunit (*n*=64) and 6.12±0.03 molecules in total for the *τ*-subunit (*n*=66). Here the error is ±s.e.m. We also verified that a YPet–β intensity standard provides the same mean intensity value under our experimental conditions (Supplementary Fig. 4g). Therefore, we subsequently use this average intensity value to estimate the number of *β*_2_-clamps in our experiments. In calculating the number of *β*_2_-molecules, we correct for photobleaching (Supplementary Note 3; Supplementary Fig. 3) and verify that the fraction of immature, dark YPet proteins is negligible (Supplementary Note 4, Supplementary Fig. 3). In our conversion from intensity to molecules, we also take into account that *β*_2_-clamps are dimers by dividing the measured YPet signal by two. This is a realistic assumption as it is believed that *β*_2_-clamps are in closed conformation even when they are not DNA-bound^22^.

Using this calibration standard, we quantify the absolute number of DNA-bound YPet–*β*_2_ molecules for individual traces of DNA replication. Representative individual time-traces of single cells clearly demonstrate that following the initiation of replication, a gradual increase of the number of DNA-bound *β*_2_ occurs until a steady state plateau is reached (Fig. 3c). This plateau is maintained until decrease is observed shortly after or during termination (Fig. 3c). From the individual traces, one can observe that there is significant cell-to-cell variability in the absolute number of DNA-bound *β*_2_-clamps, but that the overall trend in which the number of DNA-bound *β*_2_-clamps is constant for a significant fraction of the cell cycle is the same for all cells. We compared this temporal behaviour with that of a different replisome component, the *τ*-subunit of the clamp-loader in a strain in which both the *β*_2_-clamp and the *τ*-subunit are fluorescently labelled (Supplementary Note 5). The *τ*-YPet fluorescence signal fluctuates strongly and does not yield a stable plateau, in contrast to the mCherry–*β*_2_ fluorescent signal (Supplementary Fig. 6).

To obtain statistically significant values for both the total number of *β*_2_-clamps in the cell and the number of DNA-bound *β*_2_-clamps, we extracted the average behaviour from analysis over numerous cells (*n*=137; Fig. 3d,e). Figure 3d clearly depicts that for an average cell, the fraction of DNA-bound *β*_2_-clamps is more than half of the total content in the cell, which decreases to roughly zero after termination. During the cell cycle, an average cell doubles its YPet–*β*_2_ content from ~60 to 120 molecules. This number of *β*_2_-clamps in the cell is in good agreement with ensemble western estimates we performed under the same growth conditions (Supplementary Note 6; Supplementary Fig. 7). Remarkably, the number of DNA-bound YPet–*β*_2_ is held at a stable value of 
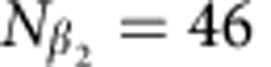
 (s.d.=12, s.e.m.=1; Fig. 3e (inset)). We also observe that the number of DNA-bound *β*_2_-clamps are close to zero before initiation and after termination. We ruled out the presence of an ectopic *dnaN* gene, by verifying that only a single copy of the *dnaN* gene is present in the strain that we used for these experiments (Supplementary Note 7; Supplementary Fig. 7). The experiment was successfully reproduced with a different fluorescent protein fusion (mCherry–*β*_2_), which strengthens the argument that accumulation is unlikely the result of fluorophore aggregation^49^,^50^, but rather due to physiological build-up of DNA-bound clamps (Supplementary Note 8; Supplementary Fig. 8). The slightly lower mean number of DNA-bound clamps (
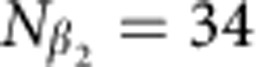
, s.d.=12, s.e.m.=1.5) as measured using the mCherry–*β*_2_ protein fusion, in combination with the mCherry intensity calibration, possibly results from the less ideal photophysical properties of mCherry, which make it less suitable than YPet for rigorous quantitative fluorescence microscopy.

### Single *β*_2_-clamps are not rapidly unloaded *in vivo*

**Figure 4:**
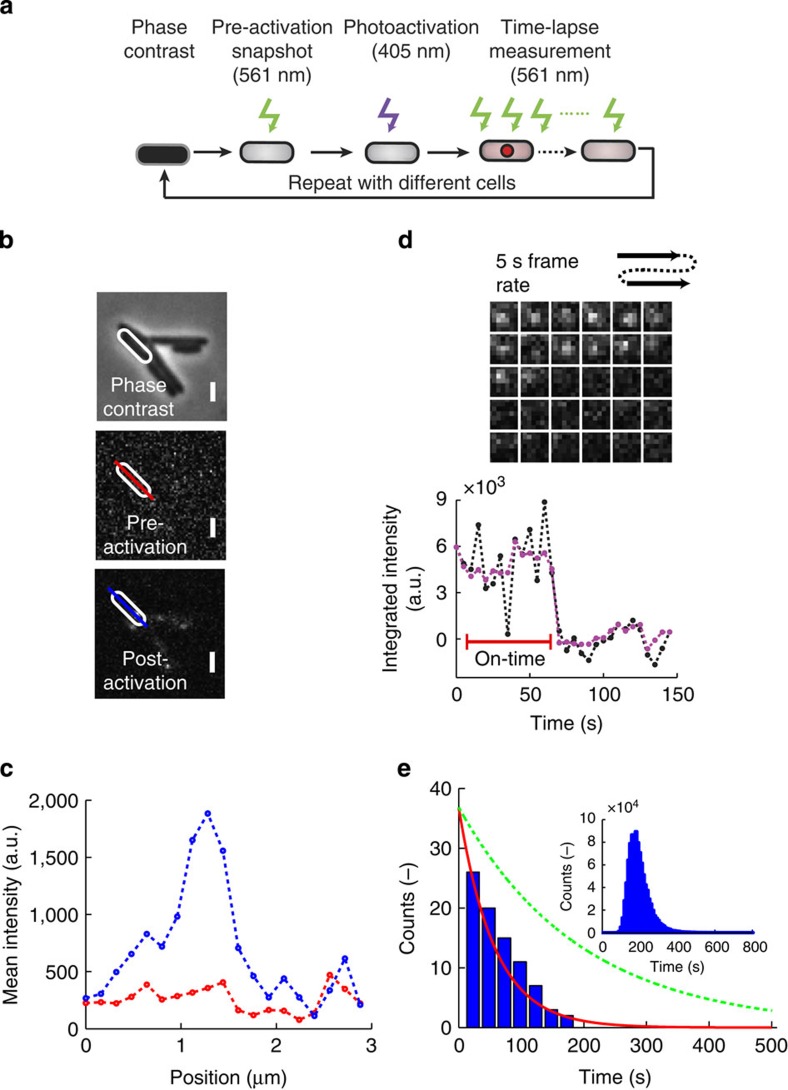
Direct measurement of the *in vivo* unloading time of the *β*_2_-sliding clamp during replication. (**a**) Illustration of the measurement sequence to image a single *β*_2_-clamp unloading event. First a phase-contrast (PH) and pre-activation snapshot are taken, after which molecules are activated only once, and subsequently imaged until foci are no longer visible (**b**,**c**) Single PAmCherry–β molecules are visualized by PALM. The sample PH image together with the respective pre-activation and post-activation fluorescence images illustrate that a single PAmCherry–β molecule can successfully be photoactivated. The corresponding pre-activation (red) and post-activation (blue) line profile plots of the single DNA-bound PAmcherry–β molecule. Scale bar, 1.6 μm. (**d**) A representative example of a montage showing the fluorescence intensity of a PAmCherry-β molecule over time and the corresponding intensity trace of the signal. The single-step disappearance is indicative of a single molecule. (**e**) On-time distribution for individual PAmCherry-β molecules (*n*=84) fitted with an exponential (red line), and the distribution for the unloading times corrected for photobleaching (dashed green line). The inset shows the distribution of the fitted unloading time constants over the 10^6^ bootstrapped data sets from which the confidence interval for the unloading time is determined.

To study the *in vivo* unloading time of an individual *β*_2_-clamp, we utilized single-molecule PALM (Fig. 4a). The endogenous *dnaN* gene was replaced with a functional N-terminal PAmCherry^51^ fusion (see Methods). Fluorescence images are acquired under shuttered 561 nm excitation (see Methods), while activation is performed once with low 405 nm laser illumination, such that on average less than one DNA-bound PAmCherry–β per cell is activated. Before fluorescence activation, a phase-contrast (PH) image is acquired to determine the cell’s position and its periphery. Sample pre- and post-activation images (Fig. 4b), together with the corresponding line-profile intensity plots (Fig. 4c), demonstrate successful activation of individual DNA-bound PAmCherry–β molecules in our strain. The advantage of PALM over more conventional techniques like Fluorescence Recovery After Photobleaching and Fluorescence Loss In Photobleaching for measuring protein turnover is that it allows one to directly image a single unloading event, as shown in the sample temporal montage and the corresponding integrated intensity trace (Fig. 4d). We image a different field of view of cells for each complete PALM measurement sequence (Fig. 4a) to ensure that the cell physiology and *β*_2_-clamp behaviour are not influenced by excessive 405 nm light exposure. Using the individual analysed traces from different cells, we are able to build up a distribution for the on-time events of single PAmCherry–β molecules (Fig. 4d). To visualize a single unloading event, we only image one out of the two PAmCherry–*β*_2_ dimer subunits. After correcting for photobleaching (Supplementary Note 9; Supplementary Fig. 9), we estimate the *in vivo* unloading time to be *t*_unload_=195±58 s per *β*_2_ (Fig. 4e). This result is in good agreement with previous *in vitro* experiments (127 s per *β*_2_; ref. 24).

### The effective *in vivo* loading rate of *β*_2_-clamps

The *in vivo* loading time of a *β*_2_-clamp during chromosomal replication provides us with insight into how frequently a new clamp, *β*_2_, is needed for processive genome duplication. We utilize both the long time lapse and the single-molecule PALM data to compute the effective loading time *in vivo* of a new *β*_2_-clamp (
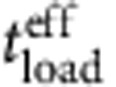
). The designation ‘effective’ is added as we do not directly measure the loading of an individual *β*_2_-clamp, but rather the total loading rate of *β*_2_-clamps onto DNA. We have shown in the preceding section that the number of DNA-bound *β*_2_-clamps remains essentially constant (
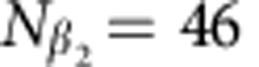
) during 2/3 of the replication process. We independently determined the *in vivo* unloading time via PALM to be *t*_unload_=195 s per *β*_2_ during the replication. In the steady-state regime, the total unloading rate of *β*_2_-clamps is balanced by the effective loading rate of *β*_2_-clamps (
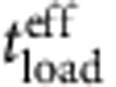
) onto newly formed primer for Okazaki fragment synthesis:


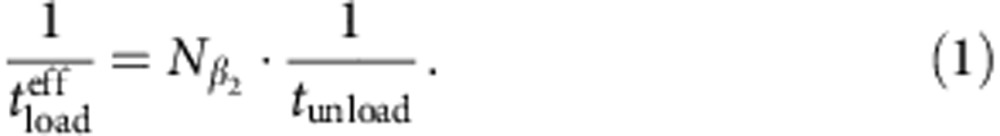


Using equation (1), we compute the *in vivo* effective loading time for a *β*_2_-clamp during the replication to be 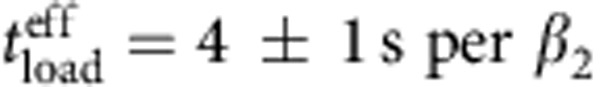. The reader is referred to Supplementary Note 10 and Supplementary Fig. 10 for a more detailed discussion of equation (1).

## Discussion

DNA replication, orchestrated by the multi-protein replisome-complex, is a process essential to cell viability. By using *in vivo* single-molecule fluorescence microscopy in combination with microfluidics, we were able to investigate the detailed dynamics of an essential component of the replisome, the *β*_2_-clamp, during DNA replication in live *E. coli* cells. Lagging-strand synthesis is a complex and highly dynamic process, and the sliding clamp is one of the key proteins involved. Each new primer-template junction requires a loaded *β*_2_-clamp to ensure processive replication by DNA Pol III, which is signalled to cycle from one Okazaki fragment to the next^52^ as the replication fork progresses at ~600 bp s^−1^. Given the average replication fork speed and the typical size of an Okazaki fragment (1–2 kb), one would expect a *β*_2_-clamp to be necessary every ~1.5–3 s. Leading-strand synthesis might be less processive than commonly believed, which would imply that a new *β*_2_-clamp would also need to be loaded on the leading strand. In what follows, however, we have assumed that during normal replication, the exchange of *β*_2_-clamps on the leading strand is a much less frequent occurrence than *β*_2_-clamp exchange for the lagging strand. Until now, it was not demonstrated *in vivo* whether these loaded *β*_2_-clamps are predominantly recycled (that is, immediately unloaded and reloaded) between successive Okazaki fragments, or whether *β*_2_-clamps remain bound to a completed fragment for a prolonged period of time.

Our results indicate that the number of DNA-bound *β*_2_-sliding clamps increases during the course of the cell cycle, peaking at more than 20 behind an individual fork. Following the initiation of replication, we observe that the number of DNA-bound *β*_2_-clamps gradually increases until a steady-state plateau is reached. This plateau, whose magnitude is such that ~50% of the total *β*_2_-clamps in the cell are DNA-bound, is maintained throughout the remainder of the cell cycle. We determined the number of *β*_2_-clamps in the cell (60–120) during replication, as well as the total number of DNA-bound *β*_2_-clamps (
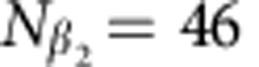
). After termination, *β*_2_-clamps are presumably no longer being loaded, and the fraction of DNA-bound *β*_2_-clamps decays accordingly.

Notably, our measurements for the number of DNA-bound *β*_2_-clamps differ from the value measured previously by some of us in a comprehensive *in vivo* study of the whole *E. coli* replisome complex^26^. In this study, the number of *β*_2_-clamps was estimated to be three for each of the two independent replisomes^46^, for a total of six DNA-bound *β*_2_-clamps present during replication. We note that stoichiometries for most of the other proteins have been duplicated independently, and, therefore, the difference of the number of DNA-bound *β*_2_-clamps appears an isolated case^27^. Although we cannot fully explain the difference in the number of DNA-bound *β*_2_-clamps, we can nonetheless delineate some possible contributions. The difference may result from the cell physiology due to the immobilization method, lower statistics due to the challenging nature of the ‘slim-field’ experiments at the time, or due to inadvertent changes of the imaging system since measurements spanned across months in the earlier study. It is thus crucial to maintain healthy cell physiology and cell cycle synchronization during experiments, which highlights the utility of microfluidics in live cell single-molecule fluorescence measurements.

The substantial number of DNA-bound *β*_2_-clamps behind each replication fork suggests that *β*_2_-clamps are not rapidly recycled during replication. To corroborate this view, we have utilized PALM to directly measure the *in vivo* unloading rate of a single *β*_2_-clamp (*t*_unload_=195 s per *β*_2_). Together with the number of DNA-bound *β*_2_-clamps in steady state, this allows us to calculate the effective time of loading (equation (1)) a *β*_2_-clamp during replication as 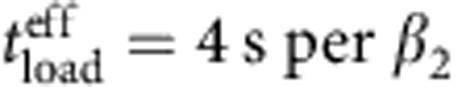. This result is in good agreement with our previously calculated average estimate of the primer formation time using the Okazaki fragment size range and the typical size of the *E. coli* genome. Also, this effective loading rate is in accordance with the model that DnaG sets the fork speed^29^. DnaG is thought to synthesize RNA primers at a rate of approximately one primer every one to two seconds^53^, which is in good agreement with our calculated *in vivo* effective loading time. We suggest that an individual *β*_2_-clamp remains on the DNA for a protracted period of time during chromosomal replication, as has been proposed on this basis of *in vitro* experiments^54^,^24^ and plasmid replication^55^. Our results are in agreement with the behaviour of the sliding clamp for the Gram-positive bacterium *B. subtilis*^34^. In this bacterium, the number of DNA-bound *β*_2_-clamps was estimated at ~200 during replication, indicative of clamps being left behind during fork progression. There is a slight possibility that the loading and unloading reaction could be sterically hampered by the fusion protein. However, we have no reason to believe that this is the case since our results are in very good agreement with previous *in vitro*^24^ and *in vivo* work^34^. Our study shows that rapid recycling of *β*_2_-clamps for subsequent lagging-strand synthesis^33^, though observed in *in vitro* experiments in the absence of excess *β*_2_-clamps in solution, is not the predominant mode *in vivo*. Although our data does not exclude that *β*_2_-clamps are rapidly recycled at the replication fork, the fact that the loading rate from solution matches the estimated primer formation rate strongly suggests that direct recycling is not the dominant mode of clamp loading.

**Figure 5:**
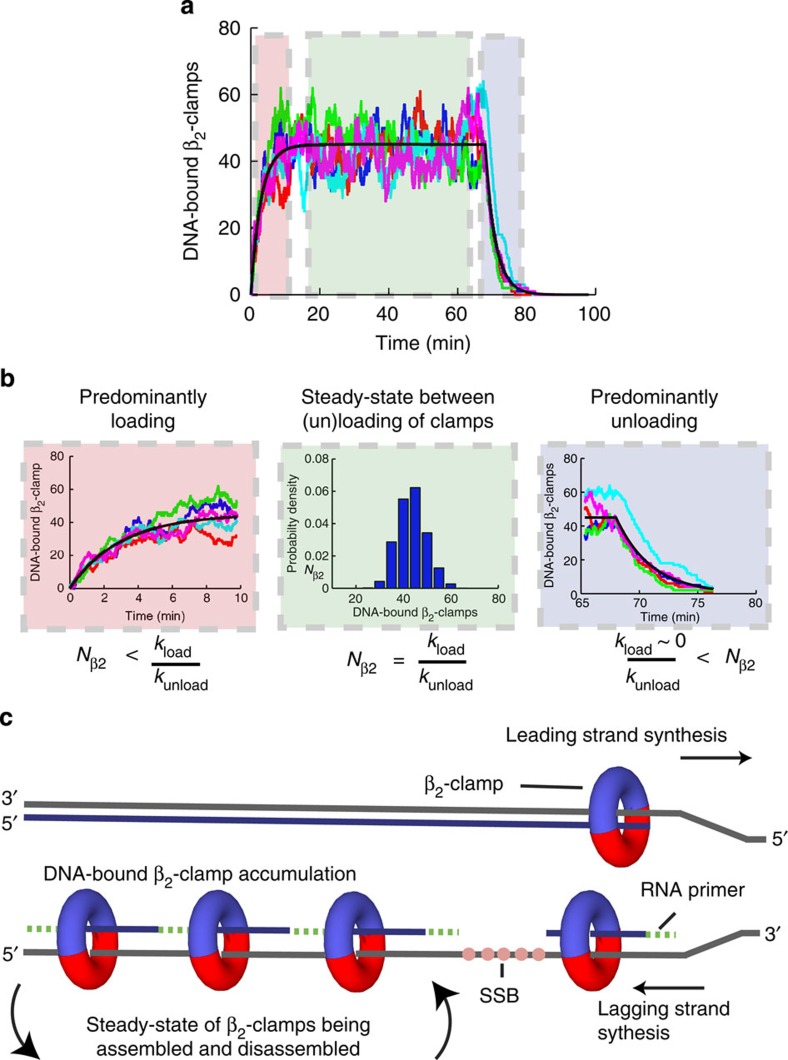
Describing the *β*_2_-sliding clamp recycling process during replication. (**a**) A Monte Carlo simulation of the *β*_2_-clamp assembly and disassembly reaction. For illustrative purposes, we perform a Monte Carlo simulation of the proposed model, utilizing the experimentally determined primer formation and unloading rate, as well as the replication time, and under the assumption that primer formation is rate limiting. We show the simulation results for five individual traces (coloured lines). The black curve is the analytical solution for the average number of loaded *β*_2_-clamps. Here we divide the total trace into three time regions, namely initiation (red), steady state (green) and termination (blue). (**b**) A zoom of the different sections from **a**. (left) A build-up of loaded *β*_2_-clamps on the DNA proceeds for ~10 min. (middle) After the gradual accumulation of loaded *β*_2_-clamps, a steady state plateau of 46 DNA-bound *β*_2_-clamps is maintained for ~2/3 of the replication process. (right) After termination, all DNA-bound *β*_2_-clamps are unloaded in ~5 min. (**c**) A cartoon illustrating the DNA-bound *β*_2_-clamp build-up during replication. As the rate at which *β*_2_-clamps are loaded (one every 4 s) is much faster than the unloading rate of individual *β*_2_-clamps (once every 195 s) during replication, there will be a dynamic reservoir of *β*_2_-clamps that have not yet been unloaded left on the lagging strand.

To illustrate the overall *β*_2_-clamp dynamics during replication, we perform a Monte Carlo simulation (see Methods) that takes our experimentally determined values for 
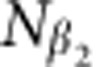
, *t*_unload_, 
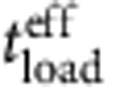
 and *t*_rep_ as input parameters (Fig. 5a). As the approximate rate of clamp removal during termination (Fig. 3e) agrees with the value measured by PALM during steady-state replication (~195 s per *β*_2_), we simply input the latter (likely more accurate) value into the simulations. The simulation starts at *t*=0 with no DNA-bound *β*_2_-clamps, after which it takes <10 min to reach a stable steady-state number of *β*_2_ bound to DNA (Fig. 5b, left). This value is maintained for ~60 min (Fig. 5b, middle), after which termination occurs and the clamps are unloaded in <5 min (Fig. 5b, right). The number of *β*_2_-clamps in steady state as well as the rise and fall times underline our measured results, and are depicted schematically in Fig. 5c.

The steady-state build-up of DNA-bound *β*_2_-sliding clamps forms a *β*_2_-landing pad^34^ for different proteins to dock themselves to DNA during the life cycle of the cell. Numerous different proteins utilize the *β*_2_-clamp via the same binding pocket^56^ to perform their respective biological function. These other *β*_2_-clamp-binding proteins range from DNA ligase for Okazaki fragment maturation, inactivation of DnaA through the *β*_2_–Hda_2_ interaction^11^,^12^,^13^,^57^,^58^, potential screening of DNA damage due to sliding capability of the *β*_2_-clamps^56^, the tethering of the necessary polymerases for repair^4^,^5^,^6^,^7^,^8^, overcoming replication barriers^59^, as well as coupling mismatch-detection and replication by positioning MutS at newly replicated DNA^60^. It is still unclear which of the above-mentioned(or other) proteins are the main users of the DNA-bound clamps that are not directly situated at the replication fork. As Okazaki fragment maturation seems to be relatively fast as assessed via Ligase and Pol I dynamics^61^, it is likely not these proteins that predominantly occupy the DNA-bound clamps. The extent to which DNA-bound *β*_2_-clamps are utilized while being docked to the Okazaki fragment will most likely be dependent on the physiological state of the cell at a particular time in its cell cycle. Under stress conditions, MutS and the different repair polymerases might predominantly make use of DNA-bound *β*_2_-clamps, whereas under minimal stress conditions, ligase CrfC and Hda_2_ are the likely candidates. A thorough *in vivo* investigation of the stoichiometry and dynamic of different *β*_2_-associated proteins over the course of the cell cycle would provide the quantitative underpinning required to provide further insight into these biological processes.

## Methods

### Strains and strain construction

All strains are derivatives of *E. coli* K12 AB1157. Strains were constructed either by P1 transduction^62^ or by λ-Red recombination^63^.

The *Ypet*–*dnaN*:*tetR–mCerulean* was constructed using P1 transduction by transducing the *YPet–dnaN* fusion^26^ together with the adjacent *kanR* gene into a strain that contains a *tetO* array (50 kb clockwise from the dif-site), as well as the chromosomal integrated chimeric gene *tetR–mCerulean*^46^. The presence of the *YPet–dnaN* gene fusion was verified using the oligonucleotides: 5′- CGTTGGCACCTACCAGAAAG -3′ and 5′- ATGCCTGCCGTAAGATCGAG -3′. The sequence of the *YPet–dnaN* fusion was confirmed by DNA sequencing.

A chromosomal fusion of the gene encoding for the photoactivatable fluorescent protein (PAmCherry1)^51^ to the N terminus of the *dnaN* gene was created using λ-Red recombination^63^. The gene encoding for PAmCherry1 was amplified by PCR. The forward primer used contains an *XmaI* restriction site (5′- GCGGGCCCCGGGATGGTGAGCAAGGGCGAGGAG -3′). The reverse primer used contains a sequence coding for an 11 amino-acid linker and a *SacI* site (5′- CGATCGGAGCTCCGCGCTGCCAGAACCAGCGGCGGAGCCTGCCGACTTGTACAGCTCGTCCATGCC -3′). The PCR product was cloned into the backbone of pROD44 (ref. 26) containing a kanamycin-resistance cassette, flanked by *frt* sites, resulting in the template plasmid PAmCherry1.

This plasmid was then used as a template plasmid for generating the insert sequence used during λ-Red recombination to create the *PAmCherry–dnaN* strain. The primer sequences used were: forward 5′- ACGATATCAAAGAAGATTTTTCAAATTTAATCAGAACATTGTCATCGTAACTGTAGGCTGGAGCTGCTTC -3′; reverse 5′- ACCTGTTGTAGCGGTTTTAATAAATGCTCACGTTCTACGGTAAATTTCATCGCGCTGCCAGAACCAGCGG -3′. The DNA fragment was gel purified and ~700 ng of the linear DNA was used for electroporation of AB1157 cells overexpressing λ-Red proteins from pKD46 (ref. 63). The correct insertion of the fragment into the chromosome of the resulting strain was assayed by PCR. The oligonucleotides used were 5′- CGTTGGCACCTACCAGAAAG -3′ and 5′- ATGCCTGCCGTAAGATCGAG -3′. The sequence of the fusion gene in this strain was confirmed by DNA sequencing.

Construction of the *mCherry–dnaN* strain. The *mCherry* gene was amplified by PCR. The forward primer used for this contains an *XmaI* restriction site (5′- TAGGCTCCCGGGATGAGCAAGGGCGAGGAGGATAAC -3′). The reverse primer used contains a *SacI* site and sequence coding for an 11 amino-acid linker (5′- AAGGAGCTCGCGCTGCCAGAACCAGCGGCGGAGCCTGCCGACTTGTACAGCTCGTCCATGCC -3′). The *Frt*-flanked kanamycin-resistant gene was amplified using the following primers: forward 5′- TTACCCGGGCATATGAATATCCTCCTTAG -3′; reverse 5′- TTAGGATCCTGTAGGCTGGAGCTGCTTCG -3′. The resulting fragment was digested with *XmaI* and *BamHI*. The *mCherry* fragment and the kanamycin fragment were cloned into pUC18 between *SacI* and *BamHI* sites.

The λ-Red recombination was performed as mentioned in the previous section using the primers: forward 5′- TATCAAAGAAGATTTTTCAAATTTAATCAGAACATTGTCATCGTAAACCTGTAGGCTGGAGCTGCTTCG -3′; reverse 5′- ACCTGTTGTAGCGGTTTTAATAAATGCTCACGTTCTACGGTAAATTTCATCGCGCTGCCAGAACCAGC -3′. The presence of the gene fusion was verified using oligonucleotides 5′- CGTTGGCACCTACCAGAAAG -3′ and 5′- ATGCCTGCCGTAAGATCGAG -3′. The sequence of the fusion gene in this strain was confirmed by DNA sequencing.

The *dnaX(*τ*)–YPet*:*mCherry–dnaN* strain was constructed using P1 transduction by transducing the *dnaX–YPet* fusion^26^ together with the adjacent *kanR* gene into a strain that contains the *mCherry–dnaN* gene fusion. The presence of the *dnaX–YPet* gene fusion after transduction was verified using the oligonucleotides: 5′- GAGCCTGCCAATGAGTTATC -3′ and 5′- GGCTTGCTTCATCAGGTTAC -3′ and similarly the *mCherry–dnaN* fusion using 5′- CGTTGGCACCTACCAGAAAG -3′ and 5′- ATGCCTGCCGTAAGATCGAG -3′. The sequences of the fusions in this strain were confirmed by DNA sequencing.

Supplementary Tables 2 and 3 provide an overview of the plasmids used, as well as a summary of the different strains. The cell morphology and the doubling times of the fusion strains in LB and M9-glycerol growth medium were compared with AB1157 wild type. No significant differences were observed (Supplementary Table 1 and Supplementary Fig. 1a). The doubling times of the cells in the microfluidic device were similar (slightly faster) compared with cells grown in a shake flask (Supplementary Fig. 2). We also confirmed that in the absence of IPTG (the experimental condition used during long time-lapse microscopy), no DNA-bound foci were detected for the *YPet–dnaN*:*tetR–mCerulean* strain (Supplementary Fig. 1b).

### M9 growth medium used in experiments

The M9 growth medium used in experiments is as follows. One litre of M9 growth medium contains 10.5 g l^−1^ of autoclaved M9 broth (Sigma-Aldrich); 0.1 mM of autoclaved CaCl_2_ (Sigma-Aldrich); 0.1 mM of autoclaved MgSO_4_ (J.T.Baker); 0.3% of filter-sterilized glycerol (Sigma-Aldrich) as carbon source; 0.1 g l^−1^ of filter-sterilized five amino acids, namely L-threonine, L-leucine, L-proline, L-histidine and L-arginine (all from Sigma-Aldrich) and 10 μl of 0.5% filter-sterilized thiamine (Sigma-Aldrich).

### Microfluidics for extended time-lapse microscopy

We use our own design^43^ of the previously published microfluidic device known as the mother machine^44^ for cell immobilization during long time-lapse experiments. The reader is referred to Moolman *et al*.^43^ for a detailed description of the complete fabrication process. Here, we only briefly outline the main steps involved. First, we use electron-beam lithography in combination with dry etching techniques to create the structure in silicon. Next, we make a negative mould of this structure in polydimethylsiloxane (PDMS). The PDMS mould is then used to fabricate the positive structure in PDMS, which is subsequently used for experiments.

### Preparation of cells for microscopy

Cells were streaked on Luria-Bertani (LB) plates containing the appropriate antibiotic. Single colonies from these plates where inoculated overnight at 37 °C with shaking in M9 medium supplemented with 0.3% glycerol (Gly), essential nutrients together with the appropriate antibiotics. The subsequent day, the overnight culture was subcultured into the same medium and grown at 37 °C with shaking until an OD_600_~0.2 was reached. Cells were concentrated by centrifugation for 2 min at 16,100 *g*. The subsequent steps are dependent on the type of microscopy experiment performed as outlined next.

For agarose pad experiments, the supernatant was decanted and the pellet was resuspended in 100 μl M9-Gly supplemented with essential nutrients. The resuspended cells were subsequently vortexed for 2 s and immobilized on an M9-Gly 1.5% agarose pad between two coverslips. (The coverslips were ultrasonically cleaned in acetone and isopropyl alcohol and burned by a flame to minimize the fluorescent background before use).

For microfluidic device experiments, the supernatant was decanted and the pellet was resuspended in 50 μl M9-Gly with essential nutrients and injected into the microfluidic device. After injection into the device, the device was centrifuged for 10 min at 2,500 *g* (Eppendorf 5810R) so as to load the cells into the growth channels. Following centrifugation, the device was mounted on the microscope with tubing attached and incubated for ~45 min at 37 °C. After incubation, fresh M9-Gly with essential nutrients and the appropriate antibiotics are flushed through the device. The syringe containing the medium is then attached to an automated syringe pump to continuously infuse fresh M9-Gly, essential nutrients and 0.2 mg ml^−1^ bovine serum albumin (BSA) through the device at a rate of 0.5 ml h^−1^.

### Microscope setup

All the images were acquired on a commercial Nikon Ti microscope equipped with a Nikon CFI Apo TIRF × 100, 1.49NA oil immersion objective and an Andor iXon 897 Electron Multiplying Charge Coupled Device (EMCCD) camera operated by a personal computer (PC) running Nikon NIS-elements software. Cell outlines were imaged using the standard Nikon brightfield halogen lamp and condenser components. The fluorescence excitation was performed using custom-built laser illumination. A Cobolt Fandango 515 nm continuous wave diode-pumped solid-state laser was used to excite YPet; Cobolt Jive 561 nm continuous wave diode-pumped solid-state laser was used to excite mCherry and PAmCherry, respectively. PAmCherry was activated by a Votran Stradus 405 nm. All the three laser beams were combined using dichroic mirrors (Chroma ZT405sp-xxr, 575dcspxr) and subsequently coupled into a single-mode optical fibre (KineFLEX). The output of the fibre was expanded and focused onto the back focal plane of the objective mounted on the microscope. Notch filters (Semrock NF03-405E, NF03-514E, NF03-561E) were used to eliminate any laser light leaking onto the camera. The emission of the different fluorescent proteins was projected onto the central part of the EMCCD camera using custom filter sets: Chroma z561, ET605/52m, zt561rdc (mCherry), Chroma z514, ET540/30m, zt514rd (YPet), Chroma zet405, ET480/40m, zt405rdc (CFP). A custom design commercial temperature control housing (Okolabs) enclosing the microscope body maintained the temperature at 37 °C. Sample position was controlled with a Nikon stage (TI-S-ER Motorized Stage Encoded, MEC56100) together with the Nikon Perfect Focus System to eliminate Z-drift during image acquisition.

### Cell lysate preparation for intensity calibration

The cell lysate used for single-molecule intensity calibration was prepared as follows. Cells were grown overnight at 37 °C with shaking in M9 medium supplemented with 0.3% glycerol (Gly), essential nutrients together with the appropriate antibiotics. The subsequent day, the overnight culture was subcultured into the same medium and grown at 37 °C with shaking until an OD_600_~0.5 was reached. The cells were collected by centrifugation at 6,000 *g* (Beckman JLA 9.1000 rotor) for 15 min. Cells were subsequently resuspended in 5 ml M9-Gly and essential nutrients. The cell suspension was French pressed (Constant Systems) twice at 20,000 p.s.i. The cell lysate was then spun down at 30,000 *g* (Beckman JA-17 rotor ) for 35 min. The supernatant was shock-frozen using liquid nitrogen and kept at 37 °C until needed.

### Data acquisition

All data acquisition was performed on the same microscope setup. Image acquisition was performed with Nikon NIS-elements software. The acquisition protocol was dependent on the type of experiment performed as outlined next.

Long time-lapse experiments were conducted as follows. The cell outlines were imaged using standard brightfield illumination. Subsequently, the sample was excited by laser excitation (515 nm) with an intensity of ~5 W cm^−2^ as calculated according to Grünwald *et al*.^64^ The exposure time was set to 80 ms. The camera gain was set to 100. Brightfield and fluorescence images were acquired every 2.5 min. Data spanning ~10 h of measurement were acquired overnight.

We conducted two types of PALM experiments. First, we determined the bleaching characteristic of PAmCherry under our experimental conditions, and second, we measured the unloading time of a single *β*_2_-clamp. PALM images were acquired as follows. First, the cell outlines were imaged by taking a single phase-contrast (PH) image using a commercial Nikon external phase ring configuration. The sample was then excited for a single frame (400 ms exposure time) by a 561 nm laser with an intensity of ~5 W cm^−2^, calculated according to Grünwald *et al*.^64^ This image was used to determine the auto-fluorescence level due to the sample before activation. Photoactivation of PAmCherry was done with a single pulse (5 s) of 405 nm with an intensity of ~2.5 W cm^−2^, calculated according to Grünwald *et al*.^64^ Subsequently, a post-activation time-lapse of images were acquired using the 561 nm laser at the same intensity at a frame rate of either ~700 ms (bleaching experiments) or 5 s (unloading experiments) with an exposure time of 400 ms per frame. Camera gain was set to 100.

### Image analysis of long time-lapse experiments

Images were analysed with custom-written MATLAB software (MathWorks). Before any analysis, we subtract the uneven background using a rolling-ball filter^65^ and subsequently corrected for illumination heterogeneity by using the previously measured laser beam profile^66^. We also align the brightfield and fluorescence signals with respect to each other with 1-pixel accuracy. X–Y drift is corrected in both the fluorescence and brightfield images by tracking a fiducial marker in the PDMS to within 1 pixel.

Each drift-corrected region of interest, consisting out of a single growth channel, is analysed individually. The brightfield images are used to determine the cell poles of all the cells in a given frame. For the fluorescence signal, a kymograph of the fluorescence signal is constructed by summation of the pixel intensities per image perpendicular to the channel direction for each frame. This results in summed intensity information as a function of time per growth channel (Fig. 2c). We make use of the generated kymographs to determine individual replication and division cycles per cell (Fig. 2d). A post-processing step is subsequently performed to eliminate cells that did not match the following selection criteria: correct cell length, sufficient growth characteristics, observation of a complete cell cycle, clear fluorescence signal that both starts and ends in a diffuse state (Fig. 2d).

The fluorescence images of the detected individual cells that pass the above selection criteria are analysed further. We base our fluorescence analysis on an image of an individual bacterium with its long axis aligned with the horizontal direction of the image. The width of the image is equal to the length of the bacterium. We fix the height of the image such that a sufficient area above and below the bacterium is included that is indicative of the auto-fluorescence of the sample. We analyse the fluorescent intensity counts of a single bacterium using the individual fluorescence kymographs of each cell (summed line-profiles) by calculating three types of image content for a specific bacterium, namely ‘background’, ‘foci’ and ‘cytoplasm’ (Supplementary Fig. 3a). In brief, we first estimate the background fluorescence from the sample using the signal outside the bacterium. We did not have to take into account auto-fluorescence from the bacterium itself, as we conducted our experiments using minimal medium, which results in negligible levels of cellular auto-fluorescence (Supplementary Fig. 3b). The intensity outside the bacterium is used for a threshold with the remaining pixels intensities being representative of the total bacterium fluorescent counts. We subsequently separate ‘cytoplasm’ and ‘foci’ signals by determining the median of the summed line profiles. The signal significantly above this value is attributed to foci, whereas the remainder (lower values) are treated as the fluorescence signal from the cytoplasm (Supplementary Fig. 3a). This results in an integrated intensity value for the foci and also for the cytoplasm.

### Image analysis of PALM experiments

PALM data was analysed using custom-written MATLAB software (MathWorks) in combination with the freely available MicrobeTracker software^67^. Before any spot analysis, the fluorescence images are subjected to illumination correction and to alignment with respect to the phase-contrast (PH) images. The resulting corrected and aligned fluorescence images are then used during further analysis.

Using the PH image, the different cells are detected in the field of view and their respective outlines are determined using MicrobeTracker. Subsequently, using the spot detection algorithm as described in Olivo-Marin^68^, the spots in each individual image of the fluorescence time-lapse series are detected, and the integrated intensity is determined by summing the pixel values of each spot^69^. The integrated intensities of the spots are followed as function of time. This results in individual time-lapse integrated intensity traces of single molecules (Fig. 3c). The cell outlines as determined previously are overlaid with the fluorescence images. Any foci that are not situated in a bacterial cell (false positives) are rejected from further analysis. Only cells that had a clear fluorescence intensity focus were analysed. This focus is indicative of DNA-bound clamps and thus DNA replication. Foci that exhibit multiple steps in fluorescence intensity are also rejected. For the remainder of the foci, the time it takes from the start of the data acquisition until spot disappearance is recorded (Fig. 3c). These calculated time differences are indicative of molecule unloading (or bleaching, depending on the time of acquisition) and analysed further as described in the following section.

### Monte Carlo simulation of *β*_2_-loading and unloading dynamics

For illustrative purposes, we perform Monte Carlo simulations (Fig. 5) starting with no clamps loaded and no primers formed (
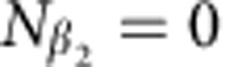
), and assuming the loading rate to be much faster than the rate of primer formation (*N*_p_≈0). In each small time-step *dt*, we let 
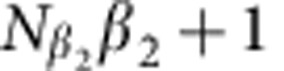
 with probability *dt* 
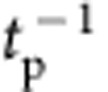
 and 
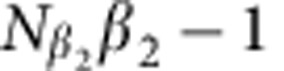
 with probability 
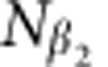
 *dt* 
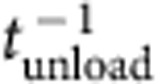
. This is repeated until the replication time is reached, upon which the primer formation rate 
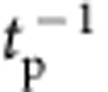
 is set to zero.

## Author contributions

M.C.M. and N.H.D. designed the research and the experiments. M.C.M. and S.T.K. undertook the experiments. S.T.K. constructed the strains. P.T. performed the western blot. S.T.K. performed the Southern blot. M.C.M. and J.W.J.K. wrote the software to analyse all the microscopy data. M.C.M., A.v.d.B. and M.D. analysed the data. A.v.d.B. performed the Monte Carlo simulation. R.R.-L. and D.J.S. provided strains and contributed to the planning and discussion of the work. M.C.M. and N.H.D. wrote the paper.

## Additional information

**How to cite this article:** Moolman, M. C. *et al*. Slow unloading leads to DNA-bound *β*_2_-sliding clamp accumulation in live *Escherichia coli* cells. *Nat. Commun.* 5:5820 doi: 10.1038/ncomms6820 (2014).

## Supplementary Material

Supplementary Information Supplementary Figures 1-10, Supplementary Table 1-3, Supplementary Notes 1-10 and Supplementary References

## Figures and Tables

**Figure 1:**
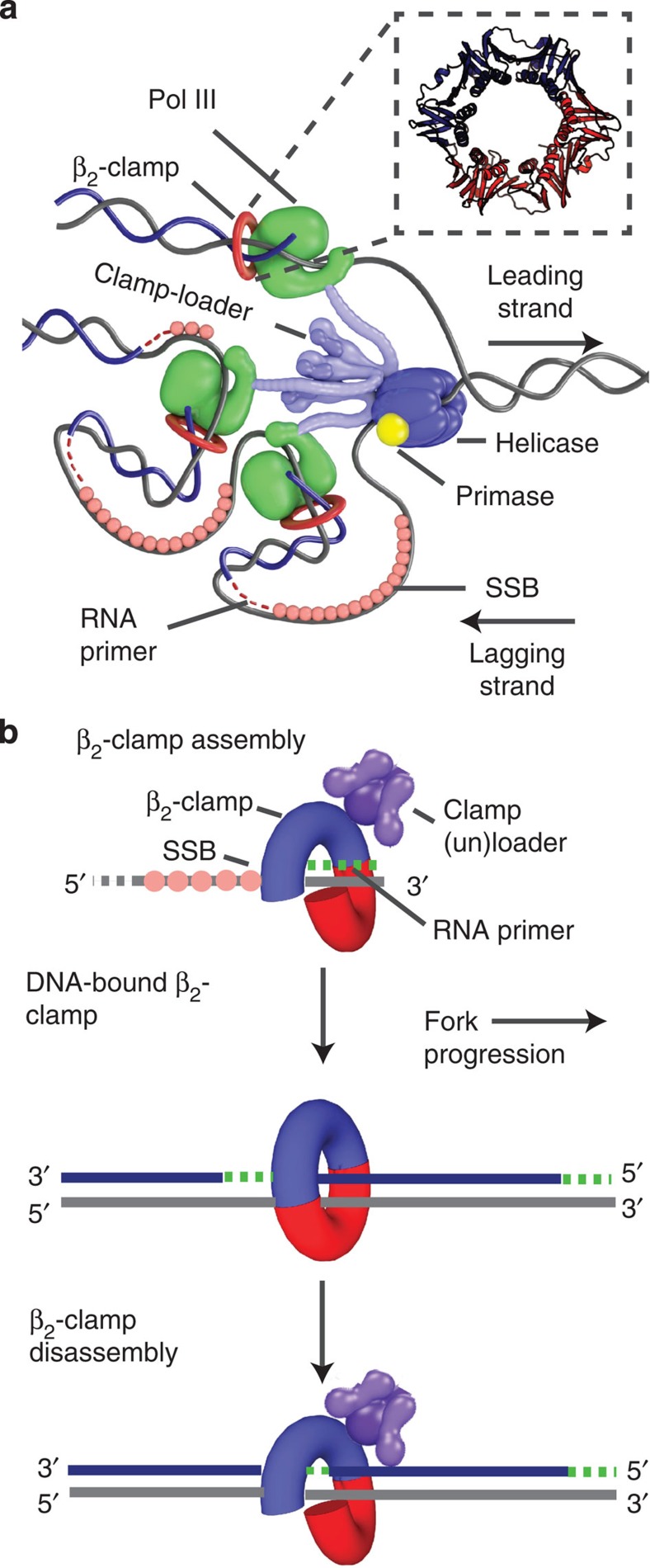
The *E. coli* replisome and *β*_2_-clamp assembly during replication. (**a**) The position of the *β*_2_-sliding clamp within the *E. coli* replisome complex. The helicase (DnaB) unwinds dsDNA ahead of the replicative polymerase (DNA Pol III), which subsequently duplicates the template strands. Different configurations of Pol IIII are potentially possible. Primase (DnaG) synthesizes short RNA primers on the lagging strand for Okazaki fragment initiation. Single-stranded binding proteins (SSB) remove the secondary structure of ssDNA and protect it from digestion. To ensure sufficient processivity during replication, Pol III is tethered to the DNA by the *β*_2_-sliding clamp. *β*_2_ is assembled onto primer-template junctions by the multi-protein ((τ/γ)_3_δδ′
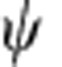
χ) clamp-loader complex. (Inset) A ribbon representation of the DNA-bound *β*_2_-sliding clamp (generated using the Protein Data Bank (PDB) file, 2POL^16^). The *β*_2_-sliding clamp is a homo-dimer that consists of six globular domains^16^. The monomers are arranged in a ring that encircles the DNA^70^ and can slide freely along it. Different proteins can bind to the two hydrophobic pockets of the *β*_2_-clamp via a conserved sequence motif^10^. (**b**) The life cycle of the *β*_2_-clamp during replication. (top) The *β*_2_-clamp is actively loaded by the clamp-loader, which opens the closed clamp and places it onto dsDNA before release^22^. (middle) The *β*_2_-clamp remains DNA-bound as long as an Okazaki fragment is being synthesized. (bottom) After the *β*_2_-clamp has reached the end of an Okazaki fragment, DNA Pol III is signalled to release^52^. The *β*_2_-clamp is believed to be disassembled by the clamp-loader.
