# Supplementary material for: Slow unloading leads to DNA-bound β2-sliding clamp accumulation in live Escherichia coli cells
Source: Nat Commun. 2014 Dec 18;5:5820. doi: 10.1038/ncomms6820 (PMC4284645; doi:10.1038/ncomms6820)
Supplement: Supplementary Information — Supplementary Figures 1-10, Supplementary Table 1-3, Supplementary Notes 1-10 and Supplementary References [file ncomms6820-s1.pdf]

SUPPLEMENTARY INFORMATION

SUPPLEMENTARY FIGURES

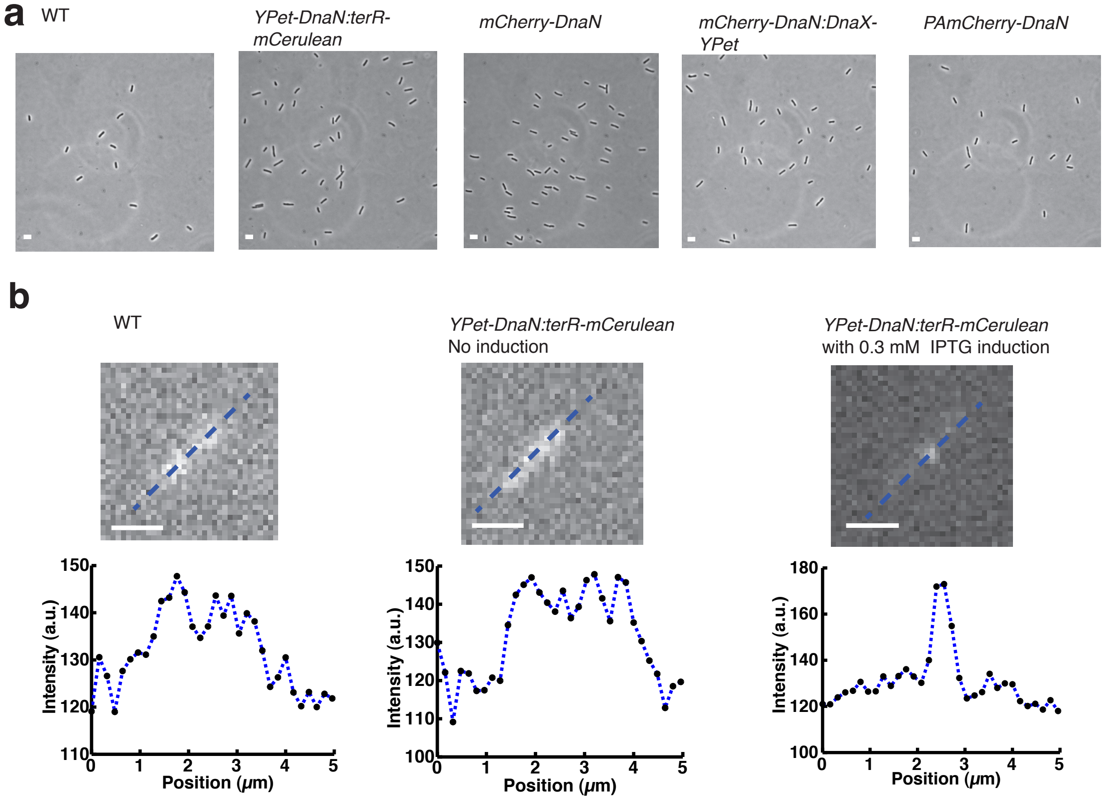

Supplementary Figure 1. Verification of the different *E. coli* variant used in this study. (a) Sample phase contrast images of the cells used in this study, which indicate that the physical appearance of these strains is essentially indistinguishable from that of WT. Scale bars: $3\,\mu m$. (b) Verification that in the absence of IPTG, terR-mCerulean is hardly expressed. Here we show the sample fluorescence images and the corresponding line-profile plots. There is essentially no difference between WT and *YPet-dnaN:tetR-mCerulean* in the absence of IPTG. Conversely, in the presence of IPTG a clear focus can be observed in *YPet-dnaN:tetR-mCerulean*. Scale bars: $1.6\,\mu m$

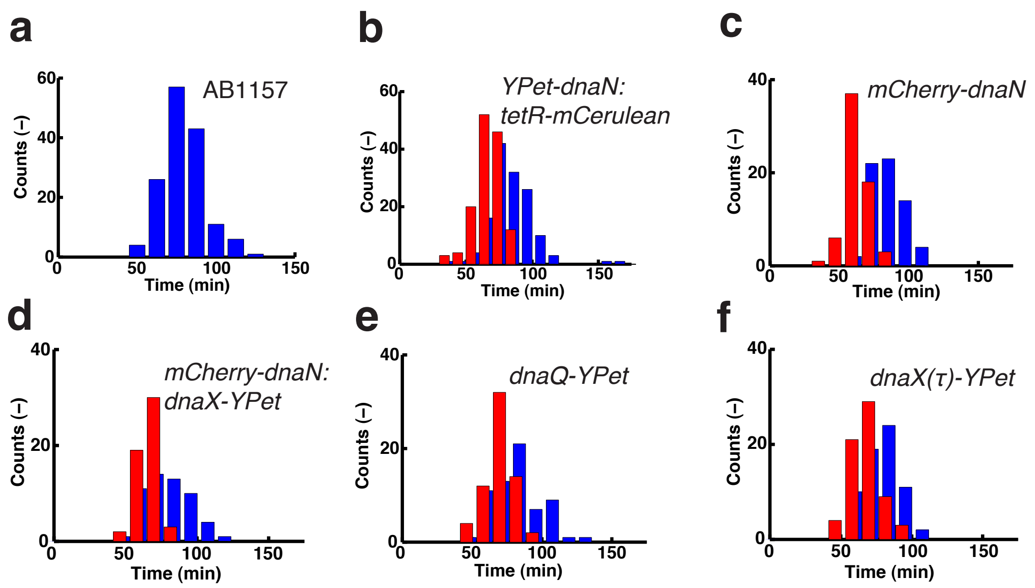

Supplementary Figure 2. Cell doubling and replication times of the different *E. coli* variants measured in the microfluidic device. (a) The doubling distribution of the AB1157 parental strain in the microfluidic device during a long time-lapse experiment. The doubling time is $t_{\text{double}} = 80 \pm 13\,\text{min}$, n = 148 (mean and ±s.d.). (b-f) The doubling (blue) and replication (red) distributions of the different *E. coli* strains in the microfluidic device during a long time-lapse experiment. The average replication time (t_{rep}) and doubling time (t_{double}) are presented with the error being ± s.d.. (b) *YPet-dnaN:tetR-mCerulean*. $t_{\text{rep}} = 68 \pm 10\,\text{min}$ and $t_{\text{double}} = 84 \pm 17\,\text{min}$ (n = 137). (c) *mCherry-dnaN*. $t_{\text{rep}} = 63 \pm 9\,\text{min}$ and $t_{\text{double}} = 84 \pm 12\,\text{min}$ (n = 65) (d) *dnaX(τ)-YPet:mCherry-dnaN*. $t_{\text{rep}} = 67 \pm 8\,\text{min}$ and $t_{\text{double}} = 79 \pm 15\,\text{min}$ (n = 54). (e) *dnaQ-YPet*. $t_{\text{rep}} = 70 \pm 10\,\text{min}$ and $t_{\text{double}} = 82 \pm 17\,\text{min}$ (n = 64). (f) *dnaX(τ)-YPet*. $t_{\text{rep}} = 67 \pm 12\,\text{min}$ and $\text{t}_{\text{double}} = 79 \pm 12\,\text{min}$ (n = 66)

.

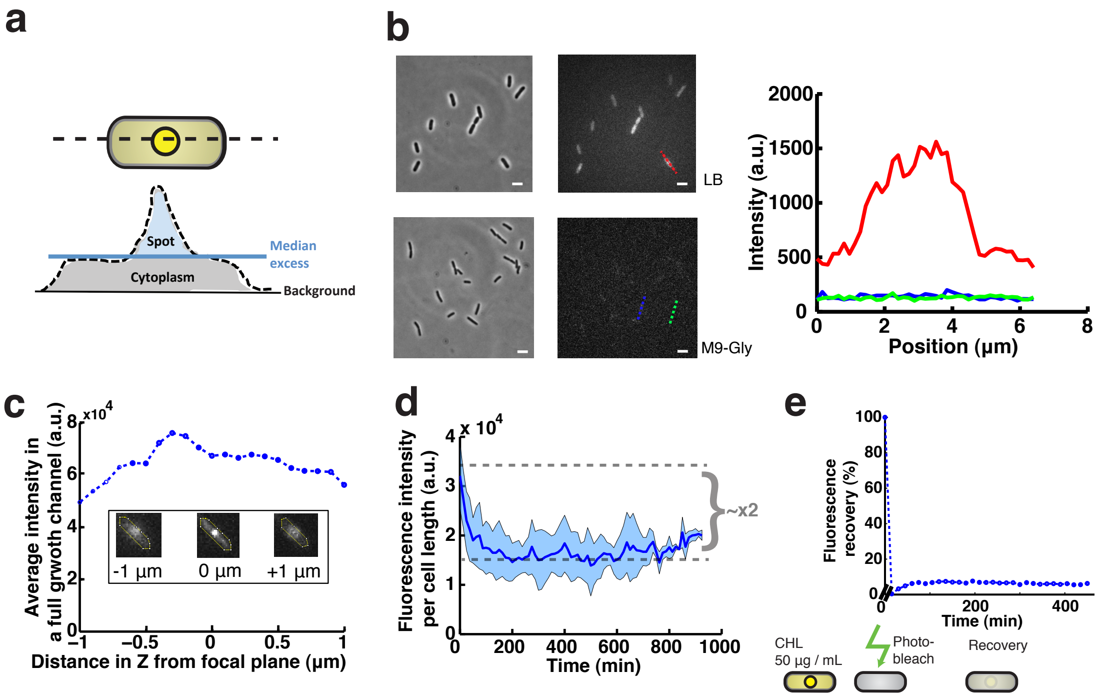

Supplementary Figure 3. The fluorescence signal definition and the different microscopy control experiments performed. a) Illustration of how the integrated line-profile is used to define sample background intensity, foci intensity, and total intensity in the cell. (b) Comparison of the degree of auto-fluorescence of *E.coli* AB1157 in LB and M9-Gly growth medium. (left) Example phase contrast and corresponding fluorescence images of cells grown in LB or M9-Gly. Scale bars: $3\,\mu m$. (right) Example line-profile plots of a cell in LB (red curve), M9-Gly (blue curve) and an arbitrary position in the M9-Gly fluorescence image (green curve). (c) A sample fluorescence signal of a z-stack of cells in a full growth channel (range: $-1\,\mu m$ to $+1\,\mu m$ with respect to the focus). (d) The bleaching behaviour of the YPet-β_2 fluorescence signal during a long-time lapse experiment. The relative change in the intensity as function of time is used to correct for YPet-β_2 bleaching. (e) Fluorescence recovery after photobleaching of cells in the presence of chloramphenicol.

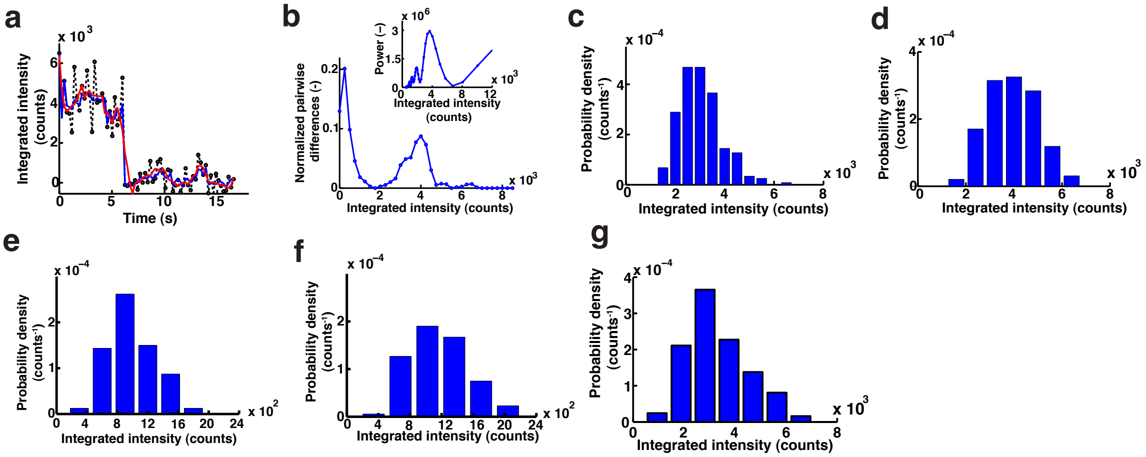

Supplementary Figure 4. Single YPet fluorescence intensity calibration. (a) A sample integrated intensity trace of a single immobilized YPet molecule as function of time. Here we show the data points (black line), the Chung-Kennedy (CK) filtered result of the data (blue line) and the Savitsky-Golay (SK) filtered trace (red). In the further analysis steps the CK-filtered data result was used. (b) The pairwise difference distribution function (PDDF) of the CK-filtered trace in (a) and its corresponding power spectrum (inset). (c) The integrated intensity distribution of single YPet molecules imaged in M9 growth medium supplemented with 0.3 % glycerol and essential nutrients (n = 235, μ = 3070, σ = 856). (d) The integrated intensity distribution of single YPet molecules imaged in french-pressed cell lysate (n = 245, $\mu = 3884, \sigma = 1108$). (e) The integrated intensity distribution of single mCherry molecules imaged in M9 growth medium supplemented with 0.3 % glycerol and essential nutrients (n = 107, $\mu = 990, \sigma = 318$). (f) The integrated intensity distribution of single mCherry molecules imaged in french-pressed cell lysate (n = 102, $\mu = 1158, \sigma = 380$). Images where acquired, for both (e) and (f), at an exposure time of 400 ms. The resulting intensity values where subsequently converted to values equivalent to an exposure time of 80 ms by dividing them by 5. (g) The integrated intensity distribution of single YPet-β molecules imaged in M9 growth medium supplemented with 0.3 % glycerol and essential nutrients (n = 131, μ = 3305, σ = 856).

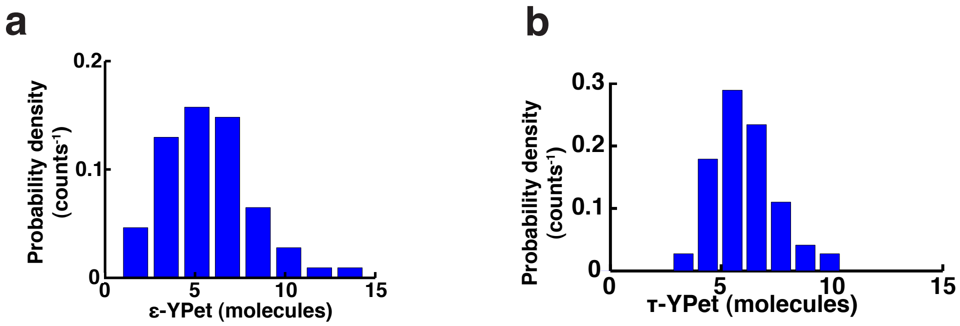

Supplementary Figure 5. Stoichiometry of DnaQ and DnaX. (a-b) The distributions of the stoichiometry of the DnaQ (ϵ-YPet) (n = 64) and DnaX (τ-YPet) (n = 66) molecules within the replisome.

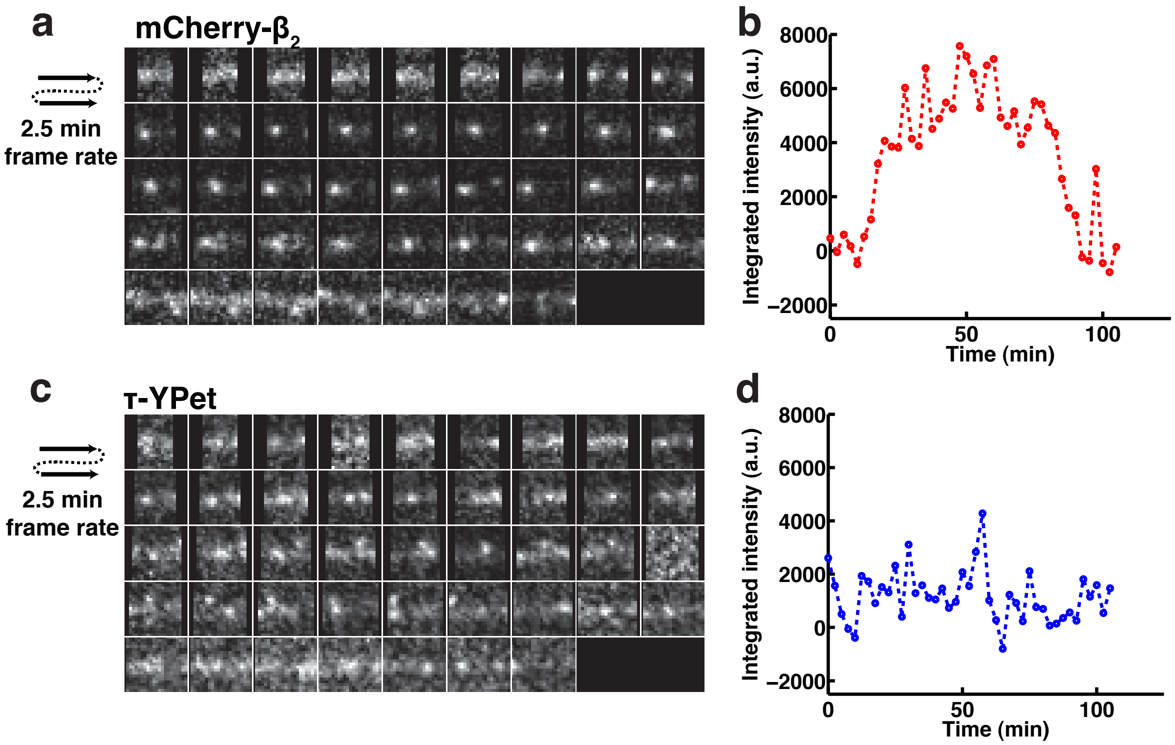

Supplementary Figure 6. The dual-color experiment that simultaneously monitors the temporal dynamics the β_2 clamp and the clamp loader (τ). (a) A sample temporal montage of the mCherry-β_2 clamp behaviour for one replication cycle. (b) The integrated intensity of the foci from the mCherry-β_2 signal shown in (a). (c) A sample temporal montage of the τ-YPet behaviour for one replication cycle. (b) The integrated intensity of the foci from the τ-YPet signal shown in (c).

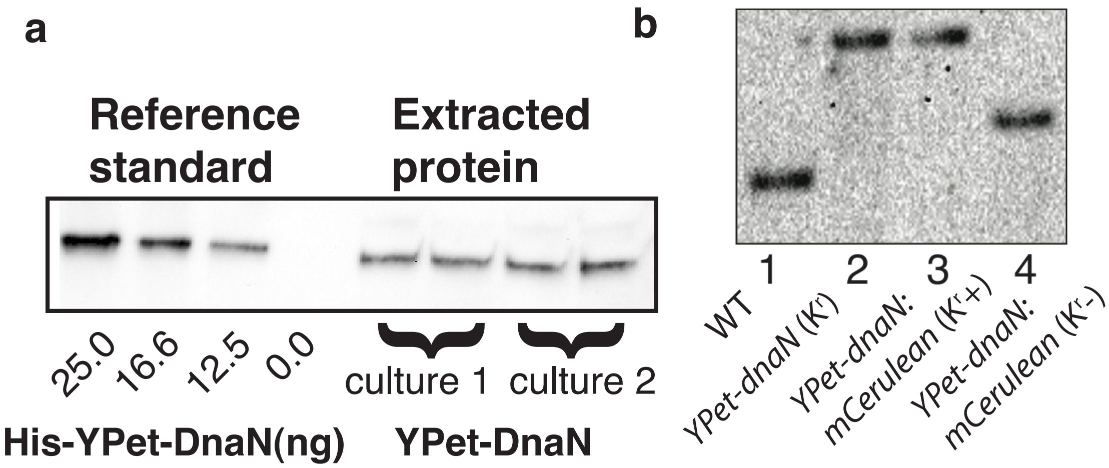

Supplementary Figure 7. Western blot and Southern blot control experiments. (a) The Western blot result, indicating the average number of clamps in the cell. Using the purified His-YPet-DnaN (Lanes 1-4 from left to right) as the quantitative standard we determined the average amount for two independent cultures (Lanes 5-8 from left to right) (b) The Southern blot result, indicating that there is only a single gene copy of *dnaN*. Here we compare WT cells (lane 1) to three different *E. coli* variant (lane 2-4) we have created. We used the strain in lane 3 (described in Methods) in this study.

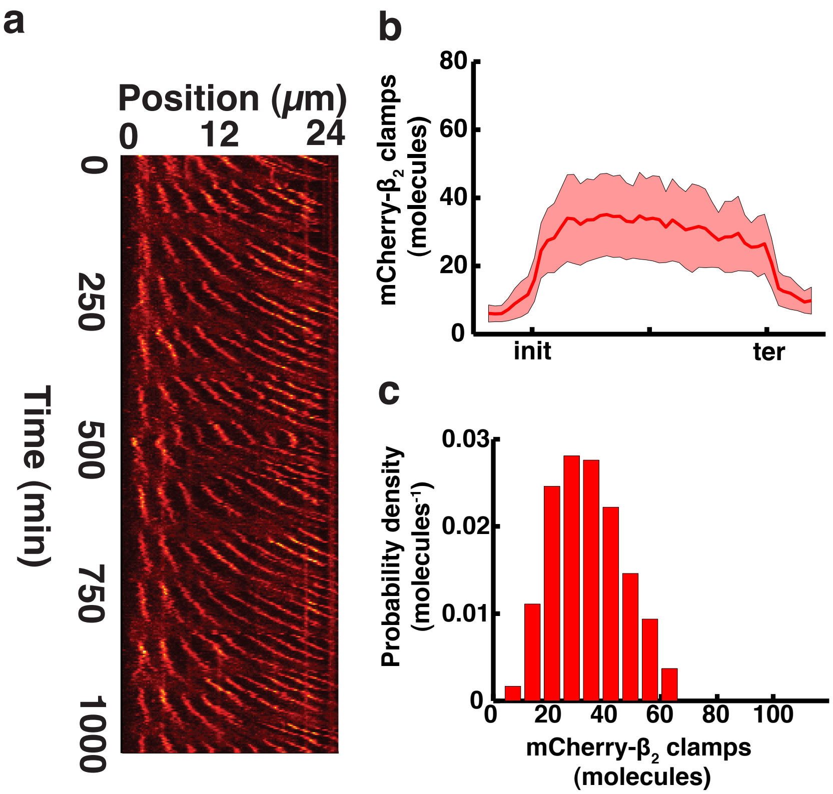

Supplementary Figure 8. The temporal dynamics of the YPet-β_2 are reproduced with a mCherry-β_2 fusion. (a) A sample kymograph of a single growth channel during an overnight experiment. (b) The behaviour of DNA-bound mCherry-β_2 clamps in both the replisomes during replication. Here we have normalized the time to make averaging of different cells, possible (n=65). The error bars represent ±s.d., from the mean. (c) The distribution of DNA-bound mCherry-β_2 clamps in both replisomes during steady-state replication. The mean number of DNA-bound clamps is $N_{\beta_2} = 34$ with s.d. $= 12$, s.e.m. $= 1.5$.

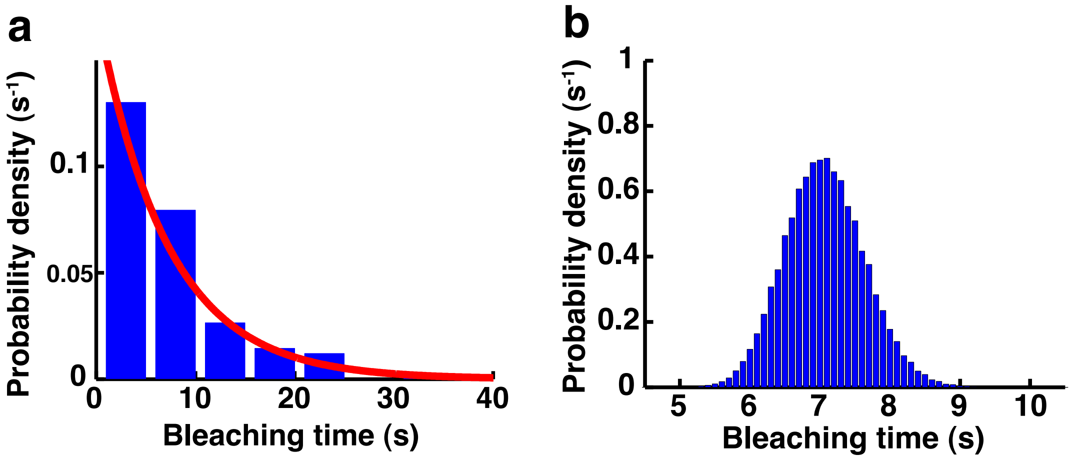

Supplementary Figure 9. The PAmCherry bleaching curve together with the bootstrapped values for uncertainty estimation. (a) The distribution of the bleaching time measured under our experimental conditions. The red curve is a single exponential MLE fit to the data. (b) The distribution of the fitted bleaching time constants over 10^6 bootstrapped data sets, from which the standard deviation for the bleaching time was determined.

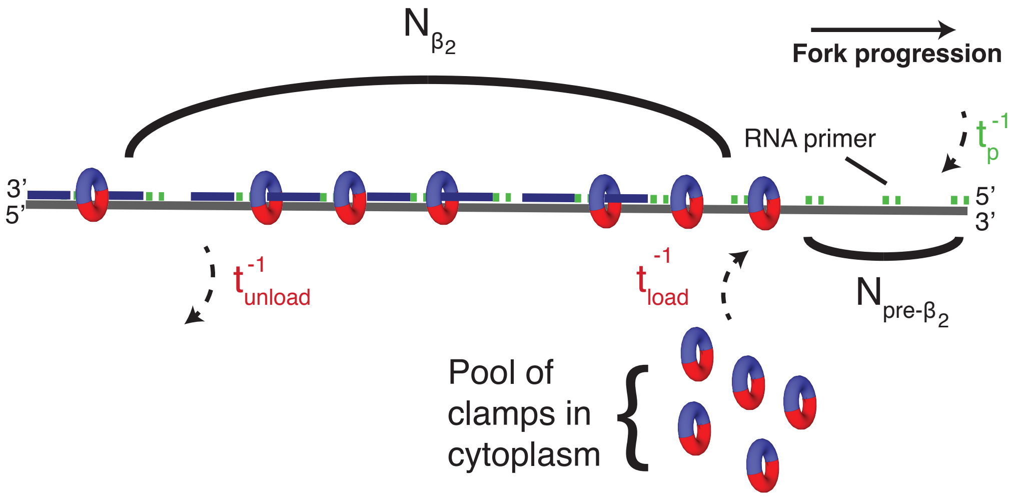

Supplementary Figure 10. A schematic illustrating the recycling model used. In our model we have five different variables. They are the number of primers having a loaded β_2 clamp (N_{β_2}), the number of primers that do not have a loaded β_2 clamp ($N_{p,unocc}$), the rate of loading a β_2 clamp (k_{load}), the rate of unloading a clamp (k_{unload}), and the rate at which primers are being formed (k_p).

SUPPLEMENTARY TABLES

Supplementary Table 1. Bulk generation times of the different strains in LB and M9-Gly growth mediumThe numbers specified indicate the mean ± s.d. The generation times where determined from three independent cultures for all the strains.

***E. coli* strain**	**Generation time (min)**	
	M9-Gly	LB
AB1157	103 ± 4	37 ± 2
YPet-dnaN:tetR-mCerulean	103 ± 1	37 ± 2
PAmCherry-dnaN	102 ± 3	38 ± 2
mCherry-dnaN	105 ± 3	38 ± 1
mCherry-dnaN:dnaX-YPet	103 ± 1	39 ± 1

Supplementary Table 2. Summary of different plasmids used in this study.

**Plasmids**	**Relevant genotype**	Construction
pKD46	Plasmid with λ-Red recombinase genes expressed under arabinose promoter	Created by standard cloning (1)
pROD44	*Ypet* template plasmid for N-terminus fusion	Cloning *Ypet* and *frt-kanR-frt* in pUC18 back-bone (2)
mCherry-N	*mCherry* template plasmid for N-terminus fusion	Cloning *mCherry* and *frt-kanR-frt* in pUC18 back-bone
PAmCherry1	*PAmCherry1* template plasmid	Cloning *PAmCherry1* in place of *Ypet* in pROD44 backbone

Supplementary Table 3. Summary of different strains used in this study.

**Strains**	**Relevant genotype**	Construction
BN1110	AB1157 strain containing pKD46 plasmid	*E. coli* K-12 derivative (3)
BN1108	*tetO* array (50 kb clockwise from the *dif*-site) and *PlacI-tetR-mCerulean* in place of *galK*	Phage transduction (3)
BN1109	*Ypet* fused to *dnaN*	λ-red recombination: *Ypet-kanR* from pROD44 $\rightarrow$ BN1107
BN1219	*YPet-dnaN* and *tetR-mCerulean*	Phage transduction: BN1109$\rightarrow$BN1108
BN1682	*mCherry* fused to *dnaN* with *kanR*	λ-red recombination: *mCherry-kanR* from mCherry-N$\rightarrow$BN1107
BN1683	*mCherry* fused to *dnaN*	*Flp-frt* recombination: *kanR* flipped out from BN1682
BN1684	*dnaX*(τ) fused to *Ypet* with *kanR*	λ-red recombination (3)
BN1864	*dnaX*(τ)-*Ypet* and *mCherry-dnaN*	Phage transduction: BN1684$\rightarrow$BN1683
BN1867	*PAmCherry1* fused to *dnaN* with *kanR*	λ-red recombination: *PAm-Cherry1-kanR* from *PAm-Cherry1*$\rightarrow$BN1107

Supplementary Notes

Supplementary Note 1

Determining the error of estimating the total YPet-β_2 content in the cell from the detected out of focus fluorescence

Using the detected fluorescence to estimate the total amount of clamps in the whole cell, we had to verify that we do not miss a significant amount of out of focus fluorescence. We verified this by determining the total intensity measured in a growth channel at different focal positions. A z-stack ($\pm 1\,\mu$m out of focus) was measured on the same growth channel. We determined the total intensity of these cells at the different Z-positions (Supplementary Fig. 3c). As can be seen, the difference is $< 10\,\%$.

Supplementary Note 2

Single-molecule fluorescence calibration

We use a similar approach as in (2, 4) to determine the average intensity of a single YPet molecule under our experimental conditions. In brief, purified single YPet molecules were immobilized on a clean cover glass through conjugation via the anti-YPet antibody (Clonetech) (5) either in M9-Gly or cell lysate. Using these two different buffer conditions, we were enable to estimate whether cytoplasm of the cell could potential influence the YPet brightness. These YPet proteins were subsequently imaged until they irreversibly photobleached using the same imaging conditions used for the long-timelapse experiments apart from a higher frame rate ($\sim 300\,$ms).

The acquired images were analyzed with custom-written MATLAB software (MathWorks). Prior to spot analyses, we subtracted the uneven background using a rolling ball filter (6, 7) and subsequently corrected for illumination heterogeneity by using a known measured laser

beam profile (8). Spot detection is performed in each individual frame for the whole time-lapse series taken using the algorithms as defined in (9). For each of the detected spots, we calculate the integrated intensity by pixel summation (10) in each individual frame. The integrated intensity of each respective spot in each frame is subsequently linked, which results in individual time-lapse integrated intensity traces (Supplementary Fig. 4a) of single molecule bleaching.

Each individual trace is subsequently processed as follows. First we perform an edge-preserving Chung-Kennedy (C-K) filter (11) on the trace, using code adapted from (12). We then calculated the pair-wise difference distribution function (PDDF) of the C-K filtered trace (Supplementary Fig. 4b). The single-sided power spectrum is subsequently calculated for the PDDF, which provided us with the intensity of that respective single YPet molecule (Supplementary Fig. 4b (inset)). Performing this analysis for subsequent YPet molecules resulted in a distribution for the intensity of a single YPet molecule in M9-Gly or cell lysate (Supplementary Fig. 4c,d) for our imaging conditions. A similar approach was utilized to calibrate the intensity of a single mCherry molecule in M9-Gly (Supplementary Fig. 4e) or cell lysate (Supplementary Fig. 4f). In both the YPet and mCherry cases, the intensity value used to convert counts to molecules is the average between the means of the M9-Gly and cell lysate measurements. We experimentally confirmed that a YPet-β fluorescence standard and a YPet standard provide the same mean fluorescent intensity in our experimental conditions (Supplementary Fig. 4g). In other words, the YPet intensity is thus unaffected by the fusion to the clamp.

As a control to verify our calibration, we determined the stoichiometry of both the ϵ-subunit (DnaQ) of DNA Polymerase III as well as the τ-subunit (DnaX) of the clamp loader complex using YPet fusions to the respective proteins. In both the cases we determined the combined stoichiometry of the proteins in both the replisomes. We reproduced the num-

bers as previously published (2) for these replisome components (Supplementary Fig. 5a,b). This established that our *in vitro* single-molecule calibration is applicable *in vivo*.

Supplementary Note 3
Estimation of β_2 clamp stoichiometry from fluorescence time-lapse data

We determine the number of sliding clamps in the cell from the integrated intensity detected using the single-molecule calibration, while correcting for photo-bleaching and converting from monomers to dimers.

To correct for photo-bleaching, we use the following approach. We assume that cell growth and protein copy number in the cell increases linearly from cell birth until cell division. The ratio of these two numbers, as function of time, should thus remain constant throughout the cell cycle of a cell if there were no photo-bleaching. However, due to photobleaching, this ratio will decrease as function of time (Supplementary Fig. 3d). We fit this curve with a single exponential and multiply the detected fluorescent signal with the appropriate factor as function of time in order to correct for this decline in fluorescence due to photobleaching.

This intensity corrected for photo-bleaching is subsequently converted into clamp dimers by dividing the fluorescence signal by the single-molecule calibration integrated intensity value as subsequently dividing by a factor of two to convert from monomers to dimers.

Supplementary Note 4
Estimation of the immature fraction of YPet molecules

We estimated the immature fraction of YPet (dark fraction) using a similar fluorescence recovery after photobleaching (FRAP) protocol as was previously published by *Badrinarayanan et al.* (13), with slight variation due to our experimental approach. An ex-

periment was prepared as described in Methods, and the cells were allowed to incubate for ~ 2 hrs without any measurement. A reference fluorescence and brightfield image were acquired after which, M9 growth medium supplemented with 50 μg/mL of chloramphenicol (CHL) was flushed through the microfluidic system. The fluorescence was subsequently bleached until roughly background level. This growth medium supplemented with CHL was continuously injected at a rate of 500 μL/hr while fluorescence and brightfield images were acquired at a frame rate of 15 min with an exposure time of 80 ms for ~ 2 hrs. The CHL treated cells do not express further proteins, and thus the fluorescence recovery is indicative of the maturation of previously (at the start of the experiment) immature YPet (Supplementary Fig. 3e). This measured value was estimated to be less than 15 % of the starting value. We estimate the maturation time of YPet in *E. coli* under our experimental conditions using the method as describe in *Wang et al* (14) to be $t_{\mathrm{mature}} < 12.5$ min.

Supplementary Note 5
Dual-color measurement of DnaX-YPet and mCherry-DnaN

In order to compare the dynamics of the clamp (mCherry$-\beta_2$) and the clamp loader complex ($\tau-$YPet) we performed a dual-color experiment. Images were acquired in the same way as for the YPet$-\beta_2$ experiment, with the only difference being measuring both the mCherry and YPet signals sequentially. As is evident from Supplementary Fig. 6, the temporal behavior of the τ-subunit is significantly different than that oft he β_2 clamp. In contrast to reaching a steady-state plateau, the τ-subunit seems to be highly dynamic and might even be exchanged during replication.

Supplementary Note 6

Determination of cellular YPet-DnaN molecules by Western blotting

His-YPet-DnaN reference standard

The cloning was conducted as follows. The *Ypet-dnaN* gene from the AB1157 cells was amplified using primers 5' – CTC CAG GGA TCC GAT GTC TAA AGG TGA AGA A – 3' and 5' – GAT CAA CAA GCT TGT GAG GGA CAT TAC AGT CTC ATT GGC ATG ACA ACA TAA GCC – 3'. The PCR amplicon was digested with *HindIII* and *BamHI*, and ligated into expression vector pRSETb (Invitrogen), resulting in construct pRSETb-dnaN. The cloning was confirmed by plasmid sequencing using the T7 promotor and terminator flanking regions. BL21pLysS cells (Promega) were transformed with the pRSETb-dnaN construct to allow inducible protein expression of YPet-DnaN-His. Bacterial cells were grown in LB broth supplemented with ampicillin (100 μg/mL) and chloramphenicol (34 μg/mL) at 37 °C with shaking until an $OD_{600} \sim 0.6$. The protein induction was performed using 1 mM Isopropyl-β-D-thio-galactoside (IPTG). After 4 hr of induction, bacterial cells were collected by centrifugation at 4000 g for 5 min. A 10 % SDS-PAGE system was used to analyze the resulting proteins.

The protein purification was performed as follows. The bacterial pellets were thawed on ice, re-suspended in buffer (50 mM Na_2HPO_4 pH 8.0, 0.3 M NaCl, 8 M urea, 10 mM imidazole) and lysed by sonication. The supernatant was cleared by ultracentrifugation at 20000 g (Beckman JA-17 rotor) for 30 min and subsequently incubated with Ni-NTA beads (Thermo) for 1 h at room temperature (RT). The beads were washed twice with 10 volumes of washing buffer (8 M Urea, 50 mM Na_2HPO_4 pH 8.0, 0.3 M NaCl 20 mM imidazole). The beads were subsequently washed with refolding buffer (50 mM Na_2HPO_4 pH 8.0, 0.3 M NaCl 20 mM imidazole) to refold the His-Tagged protein. Finally, the N-terminally His6-tagged protein was eluted from the beads using elution buffer 50 mM Na_2HPO_4 pH 8.0,

0.3 M NaCl and 250 mM imidazole). Proteins were subsequently stored in 50 % glycerol at −80 °C until used.

Culture preparation for Western blot

The *Ypet-dnaN* cells were grown overnight at 37 °C with shaking in M9 medium supplemented with 0.3 % glycerol (Gly), essential nutrients together with the appropriate antibiotics. The subsequent day the overnight culture was sub-cultured (∼ 2 %) into 50 mL of the same medium and grown at 37 °C with shaking until an OD_{600} ∼ 0.35 was reached. The bacterial culture was serially diluted in Phosphate Buffered Saline (PBS) and subsequently plated on LB Agar medium. The plates were incubated overnight at 37 °C and colony forming units (CFU) were enumerated after 24 hrs. Bacteria were harvested by centrifugation at 4000 g (Eppendorf 5810R) for 5 min. The bacterial pellet was resuspended in 2 mL of B-PER Protein Extraction Reagents (Thermo Scientific) to isolate the total amount of protein using the instructions provided by the manufacturer. Isolated proteins were subsequently stored at −80 °C until used.

Conducting the Western blot

The protein standard (25 ng, 16.6 ng, 12.5 ng of His-Ypet-DnaN) and total cell lysed were separated on 10 % SDS-PAGE gel under 100 V for 1 hr (Biorad). A Western blot assay was conducted using the above mentioned purified recombinant His-YPet-DnaN protein and total cell lysed. The gel were blotted onto a nitrocellulose membrane using a transfer buffer containing 25 mM Tris (pH = 8.3), 192 mM glycine and 10 % methanol at 200 mA for 1 hr at 4 °C. The blotted membrane was blocked with 5 % (w/v) skim milk in PBS with 0.05 % tween 20 (T-PBS) for 1 hr at room temperature. The blot was incubated for 1 hr at room temperature (RT) with the primary antibody (Clonetech). After diluting 1:10000, the blots were washed three times with T-PBS and incubated with the secondary antibodies

peroxidase conjugated goat anti-mouse IgG at a 1:7500 dilution in T-PBS. The blots were then washed three times with T-PBS and reactions were developed by SuperSignal Western Blot Enhancer (ThermoScientific) according to the manufacturer's instructions.

Quantification of bands

The bands corresponding to the protein standards, as well as bands of appropriate DnaN protein in the crude extract (Supplementary Fig. 7e) were quantified using Image Lab (Biorad). The intensities of the bands were plotted versus protein concentration (ng) and fitted to a straight line, which yielded a function to related intensity and protein concentration. This linear equation was subsequently used to calculate the amount of that protein present in the crude cell extract. Finally, the number of protein molecules per cell (C) was calculated using the following equation:

$$C = \frac{M\,A}{L\,N}. \tag{1}$$

Here M is the amount of protein in the crude cell extract (grams), A is Avogadro's number (molecules/mol), L is the molecular mass of the fusion protein ($\sim 69\,\text{kDa}$) and N is the number of cells (in the volume of crude cell extract used for Western analysis). The two independent experiments (Supplementary Fig. 7a) resulted in a combined average of 198 ± 12 YPet-DnaN monomer molecules per cell.

Supplementary Note 7

Verification of the *dnaN* gene copy number by Southern blotting

The genomic copy number of *dnaN* in WT and YPet-DnaN:tetR-mCerulean strains was verified by Southern blotting as follows. Genomic DNA was extracted from the strains. The respective DNA was digested by restriction enzymes *BamHI* and *SacI* overnight. The digested DNA samples were separated by gel electrophoresis on an 0.8 % agarose gel at 30 V

for 3 hr. The digested DNA in the gel of the strains was depurinated, denaturated, neutralized and subsequently transferred to a Hybond-N+ nylon membrane (GE Healthcare) using capillary blotting. The probe (870 bp) for southern blotting was obtained from the wildtype AB1157 chromosome by PCR amplifying the region in the *dnaN* gene using the primers 5' – GCA CCG CTA TAG GTA ACG TC-3 and 5' - TCG TCC TAC GCT ACC GAT TC – 3'. Subsequently the PCR product was labelled with thermostable alkaline phosphatase using the Amersham AlkPhos Direct kit (GE Healthcare) according to the manufacturers instructions. The labeled DNA probe was then added to the membrane and incubated at 55 °C overnight. After adding the luminescent substrate, imaging was performed using Gel-Doc. The resulting number of bands obtained is indicative of the copy number of the probed region in the chromosome (Supplementary Fig. 7b). As is evident from the blot, there is only a single band, and thus only a single copy of the *dnaN* gene, in the chromosome of the WT (lane 1) and the strain (lane 3) used in the experiments.

Supplementary Note 8
Reproducibility of the β_2 clamp dynamics with a *mCherry-dnaN* fusion

Fluorescent proteins have been show to potentially aggregate in cells. This aggregation could lead to imaging artifacts (15, 16). We conducted the same long-time lapse experiment with an mCherry-DnaN fusion in order to strengthen the argument that the stoichiometry and accumulation of clamps are reproducible with a different fluorescent protein and less likely due to any aggregation artifact. As is clearly visible the result agree within experimental error with what we have measured for the YPet-DnaN fusion strain (Supplementary Fig. 8). In determining the mCherry-DnaN stoichiometry we used the single-molecule intensity calibration done for mCherry (Supplementary Information Section).

Supplementary Note 9

Determining the unloading time of the β_2 clamp from the PALM experiments

The distribution of dwell-times of bound clamps is approximated with a single-exponential and has the following form:

$$p(t) = e^{-t/t_{\mathrm{m}}}\, t_{\mathrm{m}}^{-1}, \quad t_m^{-1} = t_{\mathrm{unload}}^{-1} + \tilde{t}_{\mathrm{bl}}^{-1}. \tag{2}$$

Here t_{m}^{-1} is the total decay rate of fluorescence, expressed as the sum of bleaching rate ($\tilde{t}_{\mathrm{bl}}^{-1}$) and the unloading rate (t_{unload}^{-1}). As we are ultimately interested in extracting the unloading time from Equation (2), we first determined the total decay rate and the bleaching rate.

To determine the decay rate of fluorescence for our experiments, the distribution in Equation (2) was fitted to the unloading data through maximum likelihood (ML) estimation, and errors were estimated over 10^6 bootstrapped data sets (Fig. 4e (inset)). This resulted in $t_{\mathrm{m}} = 57 \pm 4\,\mathrm{s}$. Here the error is ± s.d. from the bootstrapped data.

To determine the bleaching rate, we first note that in the unloading experiments the proteins are only exposed to light during a fraction of the time they stay bound. Thus, the bleaching time ($\tilde{t}_{\mathrm{bl}}$) in Equation (2) is related to the constant-exposure bleaching time (t_{bl}) through:

$$\tilde{t}_{\mathrm{bl}} = \frac{T}{t_{\mathrm{ex}}}\, t_{\mathrm{bl}}. \tag{3}$$

Here T is the experimental time between frame start times, and t_{ex} is the experimental exposure time. The constant-exposure (no shuttering, thus t_{ex} is the camera exposure in this case) bleaching time was independently determined from the unloading experiments (Fig. 9). Using ML estimation of the bootstrapped data sets, we arrive at $t_{\mathrm{bl}} = 7 \pm 0.6\,\mathrm{s}$, giving $\tilde{t}_{\mathrm{bl}} = 80.5 \pm 6.9\,\mathrm{s}$ by Equation (3). Using Equation (2), we now extract $t_{\mathrm{unload}} = 195 \pm 58\,\mathrm{s}$,

where the confidence intervals were estimated through bootstrapping 10^6 datasets (under the assumption that we can ignore the error in our estimate of the bleaching time).

Supplementary Note 10

Estimating the effective loading rate

During chromosomal replication, the number of DNA-bound β_2 clamps (N_{β_2}) fluctuates around an average in the steady state. We assume here that primers are formed at the rate t_p^{-1}, and that individual β_2 clamps are loaded at a rate of t_{load}^{-1} (Supplementary Fig. 10). The number of primers that have not been loaded with a β_2 clamp is denoted as $N_{\mathrm{pre}-\beta_2}$ and the number of DNA-bound clamps is denoted as N_{β_2}. For each cell in the steady-state regime of replication, the rate of primer formation (t_{p}^{-1}) and the total rate that β_2 clamps are loaded (${t_{\mathrm{load}}^{\mathrm{eff}}}^{-1} = N_{\mathrm{pre}-\beta_2}\, t_{\mathrm{load}}^{-1}$) must balance,

$$t_{\mathrm{p}}^{-1} = {t_{\mathrm{load}}^{\mathrm{eff}}}^{-1} = N_{\mathrm{pre}-\beta_2}\, t_{\mathrm{load}}^{-1}. \tag{4}$$

This shows that the rate of primer formation sets the effective loading rate within our model in the steady state regime. According to Equation (4), a change in primer formation rate will always be compensated by a change in the steady state value of primers that have not had a β_2 clamp loaded. Our data does not allow us to directly extract either $N_{\mathrm{pre}-\beta_2}$, or t_{load}. Similarly, if a β_2 clamp unloads at a rate, (t_{unload}^{-1}), the total effective rate at which β_2 clamps load ($N_{\mathrm{pre}-\beta_2}\, t_{\mathrm{load}}^{-1}$) must equal the total rate they unload ($N_{\beta_2}\, t_{\mathrm{unload}}^{-1}$), giving:

$$N_{\beta_2}\, t_{\mathrm{unload}}^{-1} = N_{\mathrm{pre}-\beta_2}\, t_{\mathrm{load}}^{-1}. \tag{5}$$

Together, Equations (4) and (5) give:

$$t_{\mathrm{p}}^{-1} = {t_{\mathrm{load}}^{\mathrm{eff}}}^{-1} = N_{\beta_2}\, t_{\mathrm{unload}}^{-1}. \tag{6}$$

Using Equation (6) the effective loading time is $t_{\rm load}^{\rm eff} = 4 \pm 1\,{\rm s}$. Here the error was derived via propagation from the errors in $t_{\rm load}$ and N_{β_2} and calculated from Equation (6):

$$\frac{< \Delta t_{\rm p}^2 >}{t_{\rm p}^2} = \frac{< \Delta t_{\rm unload}^2 >}{t_{\rm unload}^2} + \frac{< \Delta N_{\beta_2}^2 >}{N_{\beta_2}^2}, \quad (7)$$

where we have assumed the errors in $t_{\rm unload}$ and N_{β_2} to be uncorrelated as they originate from different experiments.

Supplementary References

[1] Datsenko, K. A. and Wanner, B. L. One-step inactivation of chromosomal genes in escherichia coli k-12 using pcr products. *Proceedings Of The National Academy Of Sciences Of The United States Of America* **97**(12), 6640–6645, June (2000).

[2] Reyes-Lamothe, R., Sherratt, D. J., and Leake, M. C. Stoichiometry and architecture of active dna replication machinery in escherichia coli. *Science* **328**(5977), 498–501, April (2010).

[3] Reyes-Lamothe, R., Possoz, C., Danilova, O., and Sherratt, D. J. Independent positioning and action of escherichia coli replisomes in live cells. *Cell* **133**(1), 90–102, April (2008).

[4] Leake, M. C., Chandler, J. H., Wadhams, G. H., Bai, F., Berry, R. M., and Armitage, J. P. Stoichiometry and turnover in single, functioning membrane protein complexes. *Nature* **443**(7109), 355–358, September (2006).

[5] Mashanov, G. I., Tacon, D., Knight, A. E., Peckham, M., and Molloy, J. E. Visualizing single molecules inside living cells using total internal reflection fluorescence microscopy. *Methods (San Diego, Calif)* **29**(2), 142–152, February (2003).

[6] Sternberg. Biomedical Image Processing. *IEEE Computer* **16**(1), 22–34 (1983).

[7] Loveland, A. B., Habuchi, S., Walter, J. C., and van Oijen, A. M. A general approach to break the concentration barrier in single-molecule imaging. *Nature Methods* **9**(10), 987–992, October (2012).

[8] Taniguchi, Y., Choi, P. J., Li, G. W., Chen, H., Babu, M., Hearn, J., Emili, A., and Xie, X. S. Quantifying E. coli Proteome and Transcriptome with Single-Molecule Sensitivity in Single Cells. *Science* **329**(5991), 533–538, July (2010).

[9] Olivo-Marin, J. C. Extraction of spots in biological images using multiscale products. *Pattern Recognition* **35** (2002).

[10] Ulbrich, M. H. and Isacoff, E. Y. Subunit counting in membrane-bound proteins. *Nature Methods* **4**(4), 319–321, April (2007).

[11] Chung, S. H. and Kennedy, R. A. Forward-backward non-linear filtering technique for extracting small biological signals from noise. *Journal of Neuroscience Methods* **40**(1), 71–86, November (1991).

[12] Reuel, N. F., Bojo, P., Zhang, J., Boghossian, A. A., Ahn, J., Kim, J., and Strano, M. S. Norse: noise reduction and state evaluator for high-frequency single event traces. *Bioinformatics* **28**(2), 296–297, January (2012).

[13] Badrinarayanan, A., Reyes-Lamothe, R., Uphoff, S., Leake, M. C., and Sherratt, D. J. In vivo architecture and action of bacterial structural maintenance of chromosome proteins. *Science* **338**(6106), 528–531, October (2012).

[14] Wang, S., Moffitt, J. R., Dempsey, G. T., Xie, X. S., and Zhuang, X. Characterization and development of photoactivatable fluorescent proteins for single-molecule-based superresolution imaging. *Proceedings Of The National Academy Of Sciences Of The United States Of America* **111**(23), 8452–8457, June (2014).

[15] Swulius, M. T. and Jensen, G. J. The helical mreb cytoskeleton in escherichia coli mc1000/ple7 is an artifact of the n-terminal yellow fluorescent protein tag. *Journal of Bacteriology* **194**(23), 6382–6386, December (2012).

[16] Landgraf, D., Okumus, B., Chien, P., Baker, T. A., and Paulsson, J. Segregation of molecules at cell division reveals native protein localization. *Nature Methods* **9**(5), 480–482, May (2012).
